# NIBAN2 Stimulates Glioma Growth by Regulating the JAK2/STAT3/c‐Myc Pathway

**DOI:** 10.1002/cam4.71239

**Published:** 2025-09-13

**Authors:** Zhi‐ming Chen, Lei Mou, Yi‐heng Pan, Chi Feng, Jun Liu, Jing‐jing Zhang, Chang‐Xiang Yan

**Affiliations:** ^1^ Department of Neurosurgery Sanbo Brain Hospital, Capital Medical University Beijing China; ^2^ Department of Neurosurgery, Hubei Provincial Clinical Research Center of Central Nervous System Repair and Functional Reconstruction Taihe Hospital, Hubei University of Medicine Shiyan Hubei China; ^3^ Department of Neurosurgery Taihe Hospital, Hubei University of Medicine Shiyan Hubei People's Republic of China; ^4^ Department of Obstetrics Taihe Hospital, Hubei University of Medicine Shiyan Hubei People's Republic of China

**Keywords:** gliomas, JAK2/STAT3/MYC, NIBAN2, proliferation

## Abstract

**Background:**

Niban‐like protein 2 (NIBAN2) has recently been linked to various neurological diseases; however, its exact role in glioma development remains unclear.

**Methods:**

Quantitative reverse transcription–polymerase chain reaction, western blotting, and immunohistochemistry were used to evaluate NIBAN2 expression in glioma tissues. In addition, we examined the effects of NIBAN2 on glioma progression in various functional trials. Animal models were used to clarify the role of NIBAN2, especially its impact on the Janus kinase 2/signal transducer and activator of transcription 3 (JAK2/STAT3) signaling pathway.

**Results:**

The research outcomes revealed that NIBAN2 was highly upregulated in gliomas and its levels were strongly correlated with tumor grade and clinical outcomes. Functional assays showed that NIBAN2 enhanced glioma cell aggressiveness by activating JAK2/STAT3 signaling and promoted tumor growth by preventing apoptosis and accelerating the cell cycle.

**Conclusion:**

The findings of this study show that NIBAN2 plays a key role in glioma aggression and poor prognosis, suggesting that it is a potential therapeutic target.

## 1 Introduction

Gliomas are the most prevalent tumors of the central nervous system (CNS). They originate from glial cells, including astrocytes, oligodendrocytes, and ependymal cells [1]. Glioblastoma (GBM) is the most aggressive tumor subtype with the worst prognosis. Although progress has been made in molecular classification, immunotherapy, and targeted therapy, the overall prognosis for patients with GBM remains extremely poor [2]. Therefore, a comprehensive investigation of the novel molecular drivers of glioma pathogenesis and the development of targeted therapeutic strategies is essential for improving the effectiveness of glioma treatment.

Niban‐like protein 2 (NIBAN2), also known as family with sequence similarity to 129 member B (FAM129B), is a member of the FAM129 family. The human gene is located on chromosome 9q34.11 and encodes a protein of approximately 85 kDa with multiple phosphorylation sites. NIBAN2 is involved in cell survival, signal transduction, and tumorigenesis [3]. It mainly regulates cell survival and adaptation to stressful environments via the PI3K/Akt/mTOR and MAPK signaling pathways. It has an important function in malignancy (including thyroid malignancy and melanoma) and certain immune‐related diseases, and is a potential biomarker and therapeutic target [4, 5, 6]. However, no studies have focused on the function of NIBAN2 in the pathogenesis of glioma. The Janus kinase 2/signal transducer and activator of transcription 3 (JAK2/STAT3) is an important signaling pathway involved in cell proliferation, differentiation, and survival and immune regulation. It is highly activated in gliomas (especially GBM) and promotes tumor progression, treatment tolerance, and immune escape [7, 8], but it is unclear whether NIBAN2 mediates its activation.

Therefore, our objectives were to investigate the function of NIBAN2 in glioma, identify novel mechanisms that may facilitate glioma progression, and propose potential targets for future therapeutic interventions.

## 2 Materials and Methods

### 2.1 Cell Lines and Reagents

Short tandem repeat DNA fingerprinting was used to validate cell lines from the Chinese National Infrastructure of Cell Line Resource (NHA, H4, U‐251, T98G, and LN‐18) and the American Type Culture Collection (HA, U118MG, A‐172, LN‐229, and U‐87MG). The cell lines were cultivated at 37°C in 5% CO_2_ and maintained in Dulbecco's modified Eagle medium (Hyclone, Logan, UT, USA) supplemented with 10% fetal bovine serum (FBS) and 1% penicillin–streptomycin (Gibco, Waltham, MA, USA). Mycoplasma screening was performed using a LookOut Mycoplasma PCR Detection Kit (Sigma‐Aldrich, St Louis, MO, USA) using previously published methods [9, 10, 11]. WP1066 (S2796) was purchased from Selleck (China). Table S1 presents detailed information regarding the antibodies used in this study.

### 2.2 Patients

Tumor samples were cryopreserved at −80°C immediately following neurosurgical glioma resections. Informed consent was obtained from all patients before surgery, and their tissues were anonymized prior to processing. The participants were notified in the consent forms that their personal data would remain confidential. This study was approved by the Human Research Committee of Shiyan Taihe Hospital and the China Anti‐Cancer Association.

### 2.3 Plasmid Constructs and Transfection

Lentiviruses encoding short hairpin RNAs (shRNAs) targeting NIBAN2 and c‐Myc (Wuhan Genecreate Biological Engineering Co. Ltd., Wuhan, China) were transduced into cells to facilitate downregulation of NIBAN2 and c‐Myc, as previously described [12, 13, 14]. The shRNA sequences targeting *NIBAN2* and *c‐Myc* are presented in Table S2. Negative control trials were performed by infecting cells with an empty vector or scrambled shRNA.

### 2.4 Cell Migration and Invasion Assays

Matrigel was added to the base of a transwell chamber with the appropriate pore size to mimic the extracellular matrix for invasion assays. Treated tumor cells suspended in serum‐free medium were placed on the upper layer at 5 × 10^3^ to 1 × 10^4^ cells/well. The lower section of the transwell chamber was filled with growth medium containing 10% FBS, and the chamber was placed in an incubator. The cell layer was then removed, and the chamber was rinsed with phosphate‐buffered saline (PBS) to eliminate any non‐migrated cells. The cells that migrated to the bottom were fixed, stained with crystal violet, and counted under a microscope.

### 2.5 Luciferase Gene Reporter Assay

To construct the test plasmid, the target gene was inserted in a reporter plasmid upstream of dual luciferase genes. A control plasmid containing an internal reference gene was used for data standardization. An appropriate cell line was cultivated to the appropriate density, and then, using an efficient transfection reagent, such as liposomes, the cells were transfected with both plasmids. To assess the activity of the target regulatory sequence, the transfected cells were treated under various conditions. After treatment, the cells were lysed to release the luciferase protein, and luciferase activity was measured using a detector. The data were normalized by dividing the target luciferase activity by the internal reference activity.

### 2.6 Cell Counting Kit‐8 (CCK‐8) Assay

During the logarithmic growth phase, the cells were resuspended and cultured at 4500 cells/well in 96‐well plates. The culture medium was inadequate and lacked necessary components for the experiments. After allowing cell adhesion and monolayer formation for 24 h, the medium was substituted, and 10 μL of CCK‐8 reagent was added to the cells. The absorbance was measured at 450 nm using a microplate reader.

### 2.7 Colony‐Formation Assay

The cells were diluted and approximately 600 cells were added to each well of six‐well plates. They were incubated at 37°C with 5% CO_2_ for approximately 2 weeks, with frequent changing of the culture medium. Following fixation with 4% paraformaldehyde for 15–20 min, the cells were stained with crystal violet for 30 min. After rinsing the dish to remove excess dye, the colony‐formation rate was determined by counting colonies with at least 50 cells.

### 2.8 Bioinformatics Analysis

Glioma gene expression data were obtained from The Cancer Genome Atlas (TCGA). cBioPortal and GraphPad Prism 10.1.2 software (GraphPad Prism Software Inc., San Diego, CA, USA) were used to perform the analyses, as previously described [13, 15].

### 2.9 Western Blotting (WB)

Cell and tissue samples were lysed, and the bicinchoninic acid method was used to quantify the total protein content. The proteins were subjected to heat denaturation, combined with a loading buffer, and separated using sodium dodecyl sulfate‐polyacrylamide gel electrophoresis. After transferring the proteins to nitrocellulose or polyvinylidene fluoride membranes, they were incubated with a primary antibody at 4°C overnight to prevent nonspecific binding. After washing, the membranes were incubated with a secondary horseradish‐peroxidase‐labeled antibody at room temperature for 2 h. The target protein signal was developed using an enhanced chemiluminescence reagent, and the band intensity was recorded using a chemiluminescent apparatus.

### 2.10 Immunohistochemical (IHC) and Immunofluorescence (IF) Staining

Tissue sections were fixed in 4% paraformaldehyde, permeabilized using 0.5% Triton X‐100, and washed. After blocking nonspecific binding using 5% BSA, the sections were incubated with primary antibodies in blocking buffer overnight and then with secondary antibodies conjugated with fluorescent dyes.

The tissue sections were counterstained with 4′,6‐diamidino‐2‐phenylindole to reveal the nuclei and were then mounted with an anti‐fade solution. High‐resolution imaging was performed using a laser‐scanning confocal microscope (FV500; Olympus, Tokyo, Japan). To conduct IHC, 4‐μm‐thick slices of formalin‐fixed, paraffin‐embedded tissue were prepared. The slices were then incubated with a primary anti‐NIBAN2 antibody and then with a Cy3‐conjugated secondary antibody. The immunoreactive score (IRS) was calculated by multiplying the staining intensity (SI) by the percentage of positive cells (PP%). The SI was rated from 0 (negative) to 4 (very strong), and the PP% was categorized from 0 (less than 1%) to 4 (more than 80%). The IRS scores ranged from 0 to 16. Ten fields from each sample were evaluated to assess the protein expression levels. Table S1 lists the antibodies used.

### 2.11 Flow Cytometry

An annexin V‐fluorescein isothiocyanate/propidium iodide (PI) apoptosis detection kit (BioLegend, San Diego, CA, USA) was used to analyze cell death. Glioma cells were cultured in six‐well plates and exposed to the specified conditions for 48 h. Following treatment, the cells were isolated using trypsinization‐free EDTA extraction, followed by two rounds of washing with cold PBS, and were resuspended in 1× binding buffer at 1 × 10^6^ cells/mL. One hundred microliters of the cell suspension was mixed with 5 μL of annexin V‐FITC and 5 μL of PI and incubated for 15 min at room temperature in the dark.

The distribution of cells throughout the cell cycle was determined. Following the procedures outlined above, glioma cells were seeded in six‐well plates. The cells were collected after treatment, rinsed with cold PBS, and fixed in 70% ethanol at −20°C overnight. After fixation, cells were rinsed with PBS and incubated with RNase A at 37°C for 30 min. Subsequently, the cells were stained for 30 min at room temperature in the dark using 5 μL of PI solution.

### 2.12 Animal Experiments

Prior to injection, 6–8‐week‐old nude mice (Beijing Vital River Laboratory Animal Technology Co. Ltd., Beijing, China) were housed in a specific‐pathogen‐free environment for 7 days. Three to five mice per group were used for separate experiments. Mouse skulls were orthotopically implanted with glioma cells from the different treatment groups at a concentration of approximately 5 × 10^5^ cells/well. The specific location of the inoculation site was 1 mm before the bregma, 2 mm lateral to the right, and 3 mm deep. The volume of injection was 5 μL. The tumor volume was assessed using hematoxylin and eosin and Ki‐67/TUNEL IF staining after euthanizing the mice 3 weeks after inoculation.

### 2.13 Statistical Analysis

Statistical analyses were performed using SPSS Statistics for Windows, version 25.0 (IBM, Armonk, NY, USA). Unpaired or paired Student's *t*‐tests were used to compare two groups, and one‐way analysis of variance (ANOVA) was used for three groups. The Shapiro–Wilk test was used to check data normality at an alpha level of 0.05. Outcomes are presented as the mean ± standard deviation or median with interquartile range. Depending on data normality and variance assumptions, a Student's *t*‐test, corrected *t*‐test, or rank‐sum test was used for two‐group comparisons. The normality of data distribution was assessed using the Shapiro–Wilk test (or Kolmogorov–Smirnov test, D'Agostino‐Pearson test, etc.). All data were subjected to normality tests. Non‐normally distributed data were analyzed using nonparametric tests, such as the Mann–Whitney *U* test or Kruskal–Wallis test. For multiple groups, one‐way ANOVA, Brown‐Forsythe ANOVA, or the rank‐sum test was used. Pairwise comparisons were corrected using Dunnett's T3 or Dunn's method to control for type I errors. A two‐sided *p* value of 0.05 was considered significant.

## 3 Results

### 3.1 NIBAN2 Was Overexpressed in Glioma and Negatively Associated With Prognosis

**FIGURE 1:**
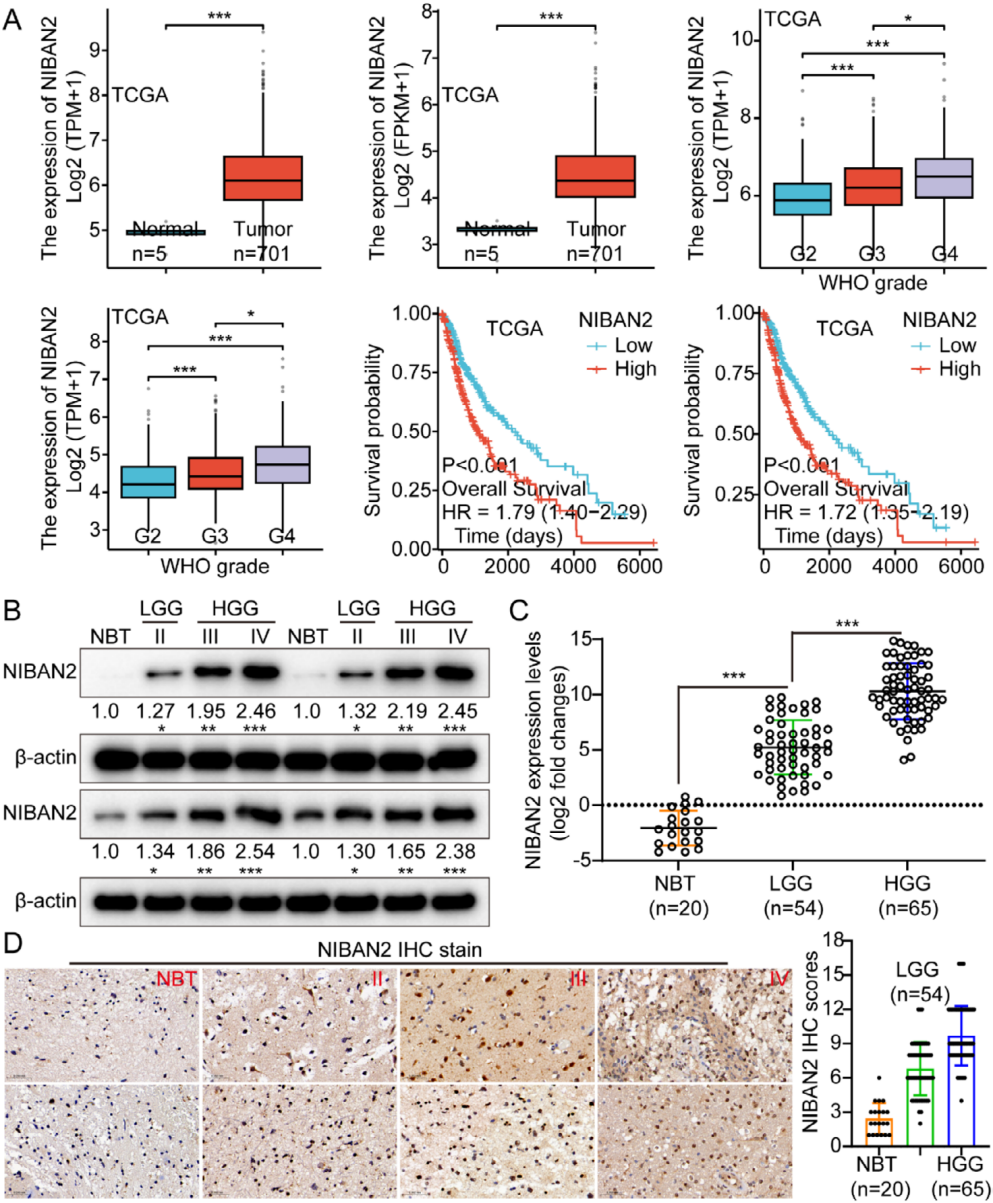
NIBAN2 was overexpressed in gliomas and negatively associated with prognosis. (A) The Cancer Genome Atlas database results demonstrated that NIBAN2 expression levels increased with increasing tumor grade, and higher levels were correlated with a negative prognosis. (B) Western blotting (WB) analysis of NIBAN2 expression in healthy brain tissue and glioma grades LGG II (low‐grade) and HGG III + IV (high‐grade). (C) *NIBAN2* mRNA levels in healthy brain tissue and diverse glioma grades assessed using quantitative reverse transcription–polymerase chain reaction (qRT‐PCR). (D) NIBAN2 concentrations in healthy brain tissue and different glioma grades were assessed using immunohistochemical staining and scoring.

We used the TCGA glioma gene expression dataset, including genomic data from five normal brain tissues (NBTs) and 701 glioma samples. NIBAN2 levels were positively associated with tumor grade and inversely correlated with patient prognosis in glioma tissues. NIBAN2 was significantly overexpressed in gliomas versus NBTs (Figure 1A and Figure S1). Herein, there is minimal difference between the two approaches, as both FPKM and TPM are standardized methods commonly used for expression quantification and grouping. The primary distinction lies in the underlying statistical assumptions employed. At present, TPM is generally considered the preferred method. Notably, the two figures essentially convey the same information. WB analysis was conducted on various cohorts of glioma tissue samples, including NBTs and gliomas categorized as grades II, III, and IV. The glioma tissues showed increased NIBAN2 levels compared to the NBTs. Moreover, there was a positive relationship between a higher glioma grade and increased expression levels of NIBAN2 at both the RNA and protein levels (Figure 1B,C). IHC analysis showed that the staining intensity of NIBAN2 varied significantly between glioma grades (Figure 1D). These findings indicate that NIBAN2 has potential as a prognostic biomarker for gliomas.

### 3.2 NIBAN2 Overexpression Promoted Glioma Cell Aggressiveness

**FIGURE 2:**
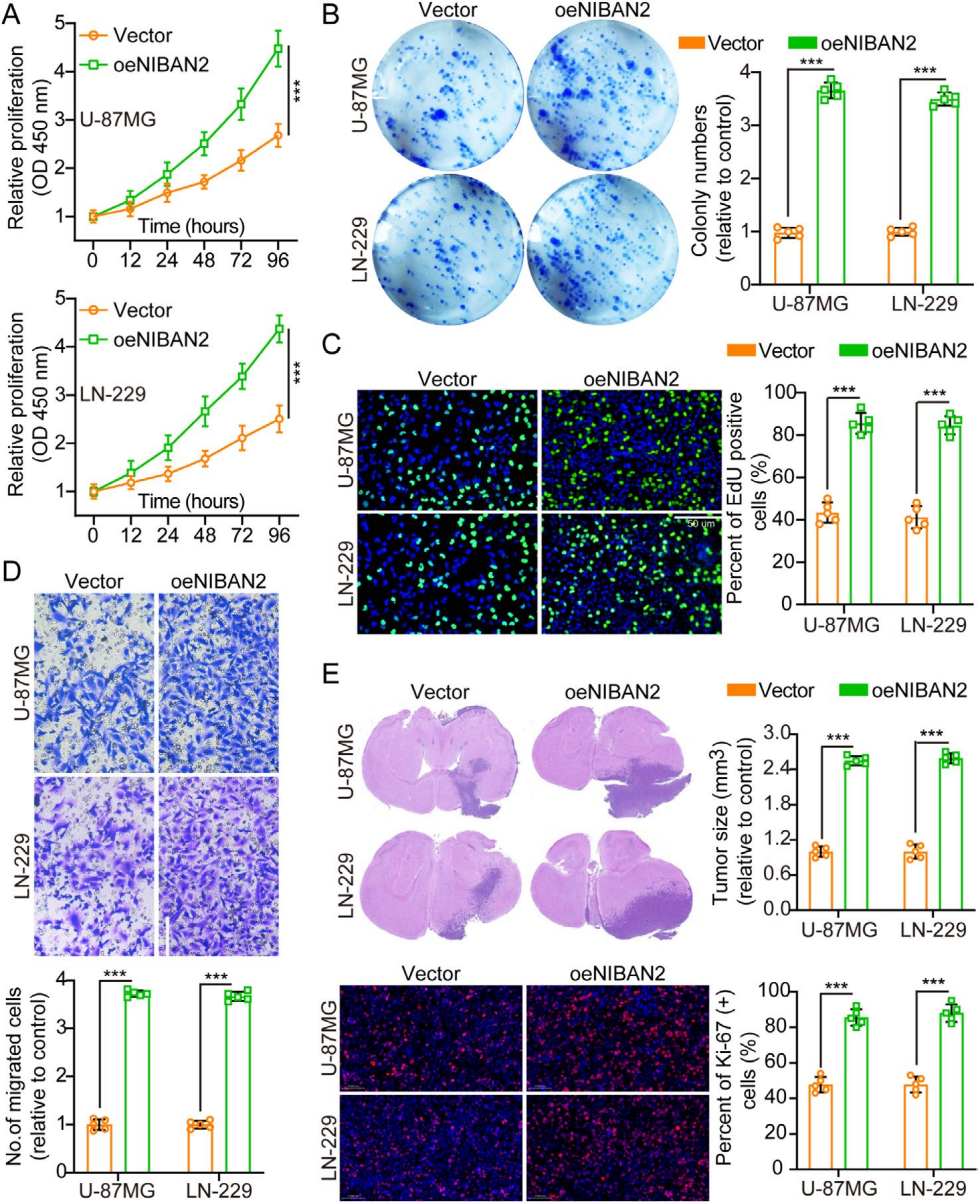
NIBAN2 overexpression promoted glioma cell aggressiveness. (A) Cell growth curves for vector and oeNIBAN2 were measured using Cell Counting Kit‐8 (CCK‐8) (*n* = 5). (B) Overexpressing NIBAN2 increased colony formation (*n* = 5). (C) EdU‐incorporation assays showed that overexpressing NIBAN2 led to an increase in the number of EdU‐positive cells, suggesting enhanced cell proliferation (*n* = 5; scale bars: 50 μm). (D) NIBAN2 upregulation increased cell migration (transwell, *n* = 5; scale bars: 20 μm). (E) Tumor size and Ki‐67 staining in the vector vs. oeNIBAN2 groups (*n* = 5).

NIBAN2 concentrations in normal human astrocytes (NHAs) and eight glioma cell lines (U‐251, T98G, LN‐229, A‐172, LN‐18, H4, U118MG, and U‐87MG) were quantified by WB. The glioma cell lines exhibited elevated NIBAN2 protein levels compared to NHAs (Figure S2A). U‐87MG and LN‐229 cells, which had moderate NIBAN2 expression levels, were selected for further studies. WB confirmed the overexpression of NIBAN2 in U‐87MG and LN‐229 cells (Figure S2B). To better understand the effect of NIBAN2 on tumor cell growth, we conducted CCK‐8, colony formation, TUNEL, and EdU assays. The results showed that NIBAN2 overexpression increased tumor cell growth (Figure 2A–C). Transwell assays showed that the overexpression of NIBAN2 significantly increased glioma cell invasion and migration (Figure 2D and Figure S2D), suggesting that NIBAN2 enhances these malignant tumor characteristics.

Flow cytometry using annexin V and PI staining was used to explore the impact of NIBAN2 overexpression. Annexin V detects apoptosis, whereas PI assesses cell cycle status. NIBAN2 upregulation decreased apoptosis and promoted cell cycle progression (Figure S3A). Additionally, NIBAN2 overexpression promoted tumor growth in vivo, with Ki‐67 IF staining showing a higher proliferation index in the NIBAN2‐overexpression group than the control group (Figure 2E). Additionally, TUNEL assays revealed that NIBAN2 upregulation reduced apoptosis, consistent with the flow cytometry findings (Figure S2F). Overall, NIBAN2 overexpression reduced cell death and accelerated tumor progression, both in vitro and in vivo.

### 3.3 Knocking Down NIBAN2 Inhibited Glioma Cell Aggressiveness

**FIGURE 3:**
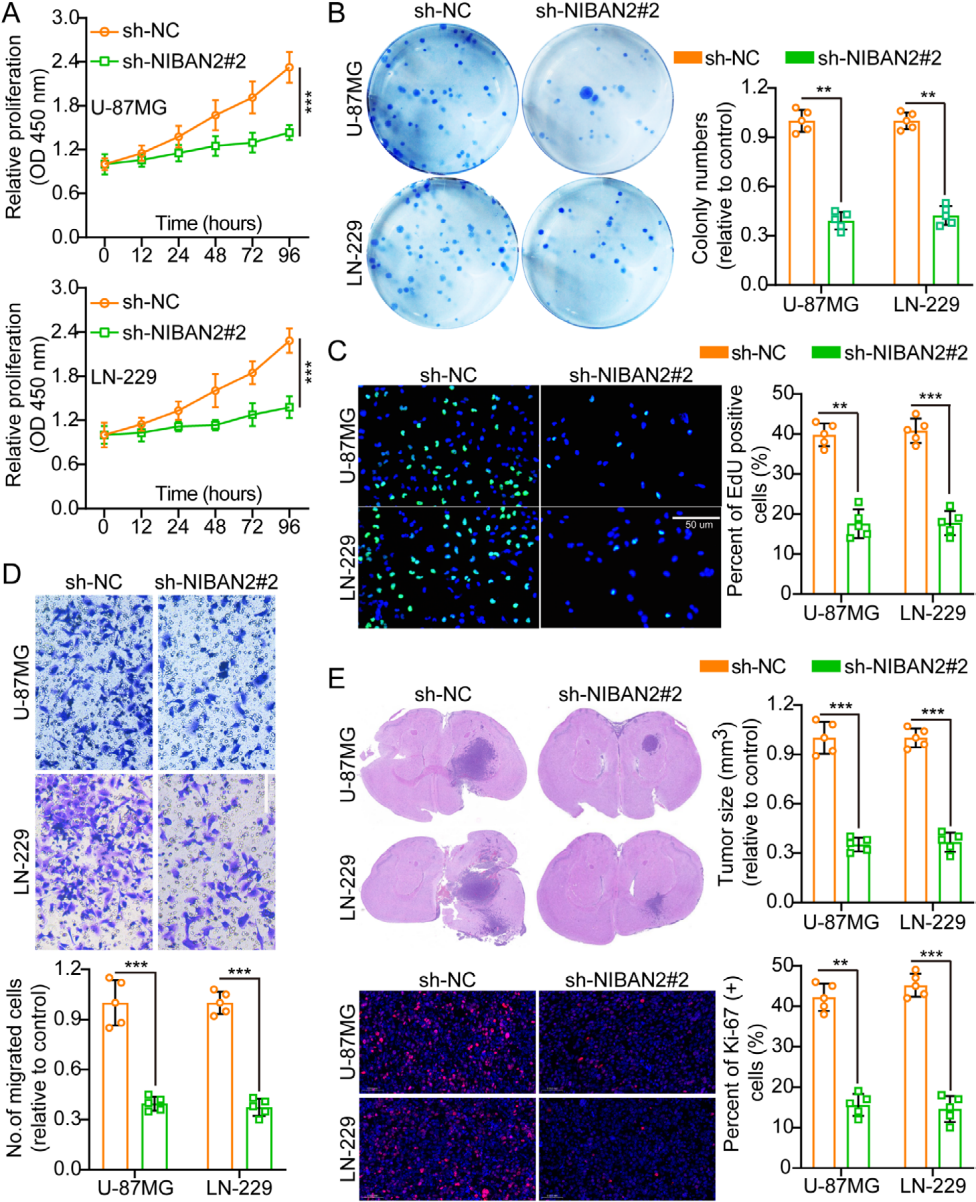
Knocking down NIBAN2 inhibited glioma cell aggressiveness. (A) Growth curves of cells transfected with sh‐NC and sh‐NIBAN2#2 were determined using CCK‐8 assays (*n* = 5). (B) Colony development was reduced after NIBAN2 knockdown (*n* = 5). (C) The EdU‐incorporation assays suggested that knocking down NIBAN2 led to a reduction in EdU‐positive cells, implying a decrease in cell proliferation (*n* = 5; scale bars: 50 μm). (D) NIBAN2 knockdown impairs migration (transwell, *n* = 5; scale bars: 20 μm). (E) Tumor size and Ki‐67 staining in sh‐NC‐ vs. sh‐NIBAN2#2‐transfected cells (*n* = 5).

WB analysis confirmed NIBAN2 knockdown in U‐87MG and LN‐229 cells (Figure S2C). Among the shRNAs tested, sh‐NIBAN2#2 showed the highest effectiveness and was used in subsequent experiments. Functional assays, such as CCK‐8, colony formation, EdU, and transwell assays, demonstrated that NIBAN2 knockdown significantly decreased glioma cell aggressiveness (Figure 3A–C and Figure S2E), indicating that NIBAN2 plays a key role in these processes. Flow cytometry showed that NIBAN2 knockdown triggered apoptosis and hindered cell cycle progression in glioma cells (Figure S3B). Cell lines with stable NIBAN2 knockdown were implanted into nude mice. After 3 weeks, the mice were euthanized and their brains were collected. IF for Ki‐67 in brain tumors revealed that NIBAN2 suppression decreased Ki‐67 levels compared to their levels in controls (Figure 3E). TUNEL assays revealed that NIBAN2 overexpression decreased apoptosis, whereas NIBAN2 knockdown increased it, consistent with the flow cytometry findings (Figure S2F). These findings suggest that NIBAN2 suppression decreased cellular proliferation in vivo.

### 3.4 NIBAN2 Activated JAK2/STAT3 Signaling

**FIGURE 4:**
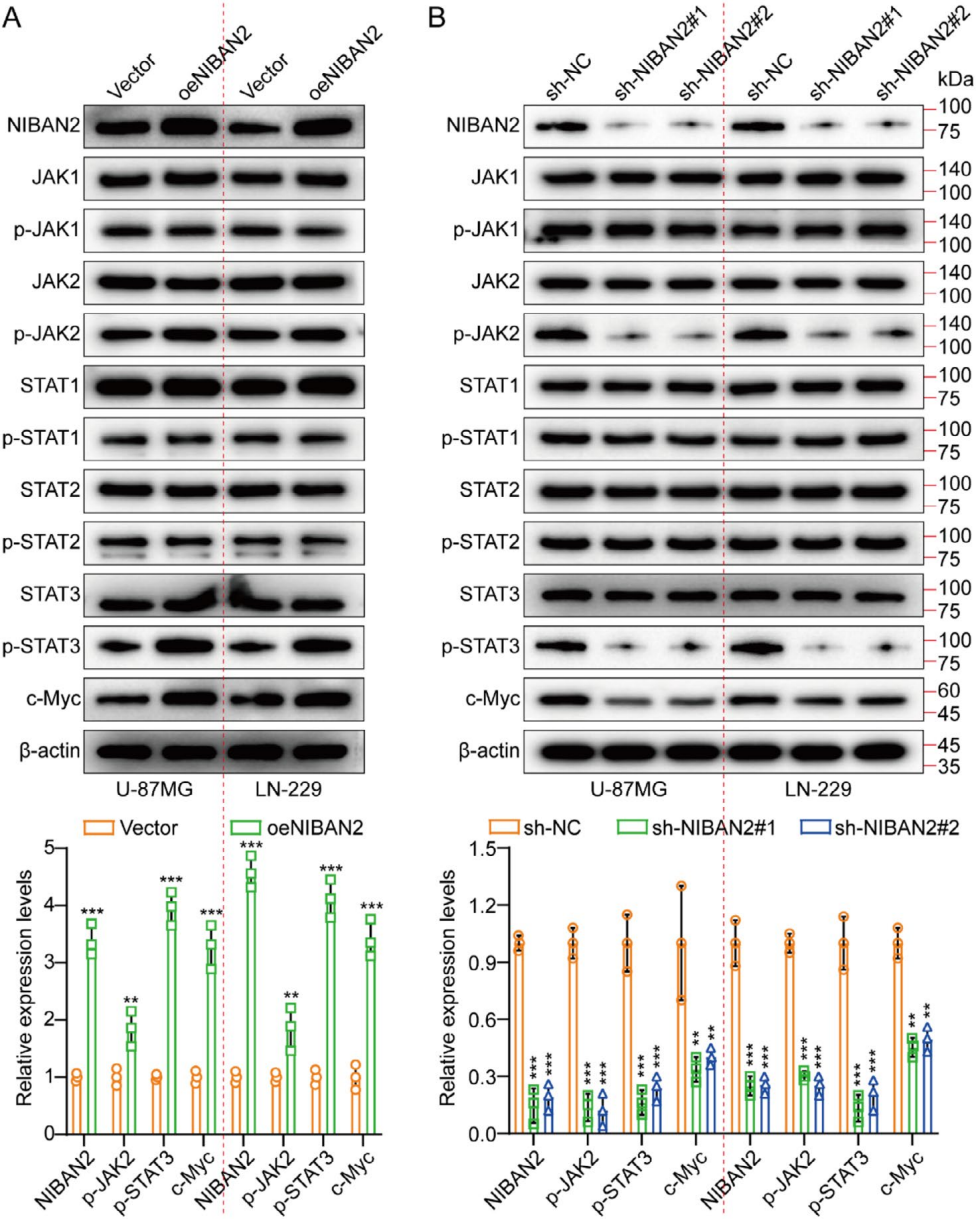
NIBAN2 activated JAK2/STAT3 signaling. (A, B) WB analysis of NIBAN2, JAK/STAT pathway components, and c‐Myc in vector, oeNIBAN2, sh‐NC, sh‐NIBAN2#1, and sh‐NIBAN2#2 groups.

Screening for possible signaling pathways using the Cignal Finder Cancer 10‐Pathway Reporter Array demonstrated that the JAK/STAT signaling axis was markedly downregulated after NIBAN2 knockdown in U‐87MG and LN‐229 cells (Figure S4A). Moreover, gene set enrichment analysis (GSEA) established a significant relationship between NIBAN2 and the JAK/STAT signaling pathway (Figure S4B,C). Moreover, to confirm the function of NIBAN2 in triggering tumor growth via activation of the JAK/STAT pathway, NIBAN2 was suppressed and upregulated in U‐87MG and LN‐229 cells, respectively. Western blot analysis of JAK1, phosphorylated JAK1 (p‐JAK1), JAK2, p‐JAK2, STAT1, p‐STAT1, STAT2, p‐STAT2, STAT3, p‐STAT3, and c‐Myc revealed that NIBAN2 suppression markedly reduced the levels of p‐JAK2, p‐STAT3, and c‐Myc, whereas RNF122 overexpression increased their level (Figure 4A,B). TCGA data analysis revealed a positive correlation between NIBAN2 levels and the levels of the signaling molecules JAK2, STAT3, and c‐Myc (Figure S5A). Collectively, these results indicated that NIBAN2 activated the JAK2/STAT3/c‐Myc pathway.

### 3.5 NIBAN2 Promoted Glioma Growth by Activating JAK2/STAT3 Signaling

**FIGURE 5:**
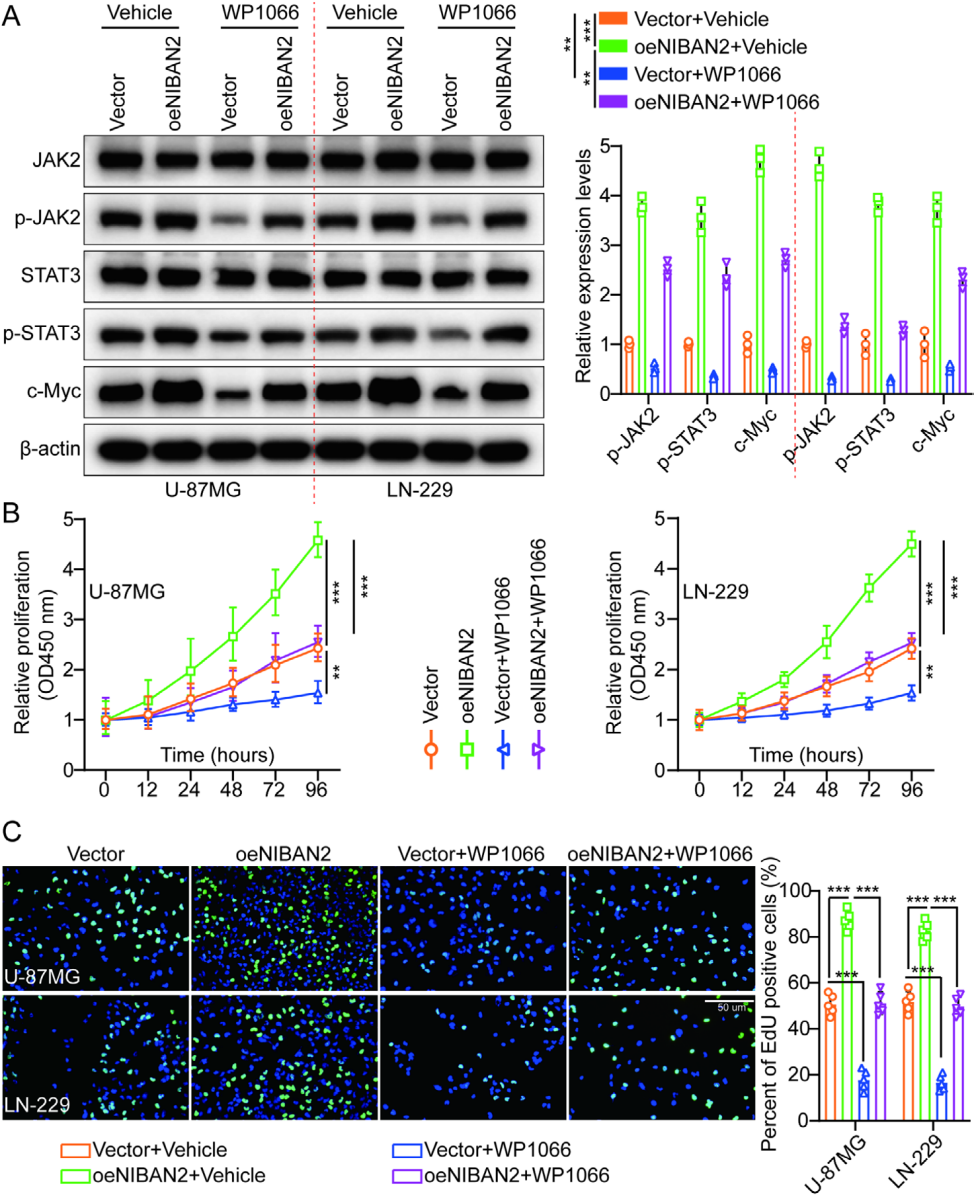
NIBAN2 triggered glioma growth by stimulating JAK2/STAT3 signaling. (A) JAK2, p‐JAK2, STAT3, p‐STAT3, and c‐Myc levels were evaluated using WB in the vector, oeNIBAN2, vector + WP1066, and oeNIBAN2 + WP1066 groups. (B) Cell growth curves for different treatment groups were determined using the CCK‐8 assay. (C) EdU assays and histogram quantification were used to evaluate cell growth across different treatment groups (*n* = 5; scale bars: 50 μm).

To further elucidate the function of NIBAN2 in promoting cell proliferation via JAK2/STAT3 pathway activation, U‐87MG and LN‐229 cells were subjected to the JAK2/STAT3 inhibitor WP1066 (6 μM, 48 h) and the effect of NIBAN2 overexpression on cell proliferation was investigated. Consistent with previous findings, NIBAN2 overexpression led to increased p‐JAK2, p‐STAT3, and c‐Myc levels, but not total JAK2 or STAT3 levels. However, this effect was attenuated when U‐87MG and LN‐229 cells were treated with WP1066 (Figure 5A). In addition, CCK‐8, colony formation, EdU, and transwell migration/invasion assays, as well as in vivo animal experiments, showed that WP1066 significantly counteracted the effects of NIBAN2 in triggering the aggressiveness of U‐87MG and LN‐229 cells (Figure 5B,C, Figures S5B and S6A,B). Consistent with the in vitro studies, these results demonstrated that WP1066 treatment (Specifically, WP1066 was administered via intraperitoneal injection (i.p.) at a dose of 40 mg/kg, once daily (QD), with the vehicle composed of 10% DMSO + 40% PEG300 + 5% Tween‐80 + 45% saline (v/v/v/v). The injection volume was 10 mL/kg (0.2 mL for a 20 g mouse). Depending on tolerability, a range of 30–60 mg/kg or alternate‐day dosing (QOD) was used as a reduced‐intensity option. Treatment was initiated on day 3 postimplantation and continued for 14–21 days.) significantly reduced tumor growth in mice with cerebral orthotopic transplantation of gliomas. Mice treated with WP1066 had lower Ki‐67 expression levels in xenograft tumors, suggesting that WP1066 may mitigate the effect of NIBAN2 on cell growth (Figure S6C). The results of the TUNEL assay further confirmed the above conclusion (Figure S7A). NIBAN2 may promote glioma growth by activating the JAK2/STAT3 signaling pathway.

### 3.6 NIBAN2 Stimulated Glioma Progression via JAK2‐STAT3‐c‐Myc Signaling Activation

Normal cellular function relies on c‐Myc proteins, which regulate the expression of genes associated with the cell cycle, proliferation, and death [16]. Mutations in or overexpression of c‐Myc genes disrupt cell growth regulation and are crucial for cancer development [17]. MYC is often overexpressed in various cancers [18, 19]. In U‐87MG and LN‐229 cell lines, shRNAs targeting c‐Myc were used to explore its function in NIBAN2‐driven glioma cell growth. Sh‐c‐Myc#2 showed the most effective knockdown and was used in subsequent experiments (Figure S7B). WB analysis showed that NIBAN2 overexpression increased c‐Myc levels, whereas WP1066 reduced this effect (Figure 5A), indicating that NIBAN2 affected c‐Myc expression via JAK2/STAT3 signaling.

**FIGURE 6:**
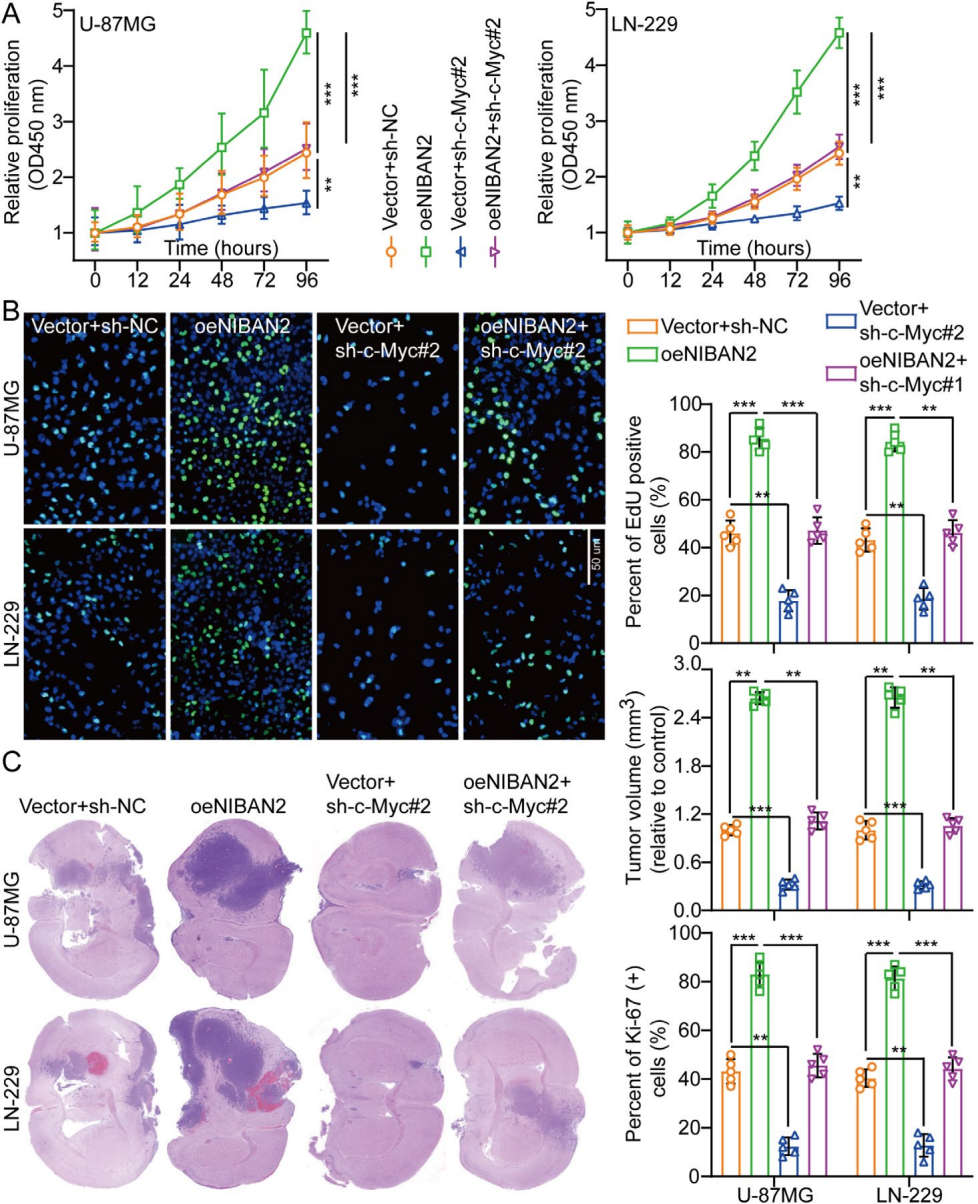
NIBAN2 stimulated glioma progression via JAK2‐STAT3‐c‐Myc signaling activation. (A) CCK‐8 assays were employed to evaluate cell growth curves in various treatment groups (*n* = 5). (B) EdU assay and histogram analysis were used to assess cell growth across various treatment groups (*n* = 5; scale bars: 50 μm). (C) Histograms of tumor weight and frozen slices of mouse brain tissue for each treatment group (*n* = 5).

Suppression of c‐Myc also reduced the proliferative, migratory, and invasive effects of NIBAN2 overexpression in the U‐87MG and LN‐229 cell lines (Figure 6A,B, Figures S7C,D and S8A). Consistent with the in vitro findings, in mice with intracranial orthotopic glioma transplantation, c‐Myc silencing significantly decreased the increase in cell growth resulting from NIBAN2 upregulation (Figure 6C). c‐Myc silencing successfully mitigated the effect of NIBAN2 on cell proliferation, as shown by the decreased Ki‐67 expression levels in xenograft tumors in the c‐Myc‐silenced group (Figure S8B). Additionally, TUNEL assay results demonstrated that NIBAN2 upregulation suppressed apoptosis, whereas c‐Myc knockdown mitigated this effect (Figure S8C). Collectively, these results indicated that NIBAN2 triggered glioma progression by stimulating the JAK2/STAT3/c‐Myc signaling pathway.

### 3.7 NIBAN2 Was Directly Targeted by Hypoxia‐Inducible Factor 1‐Alpha (HIF‐1α)

**FIGURE 7:**
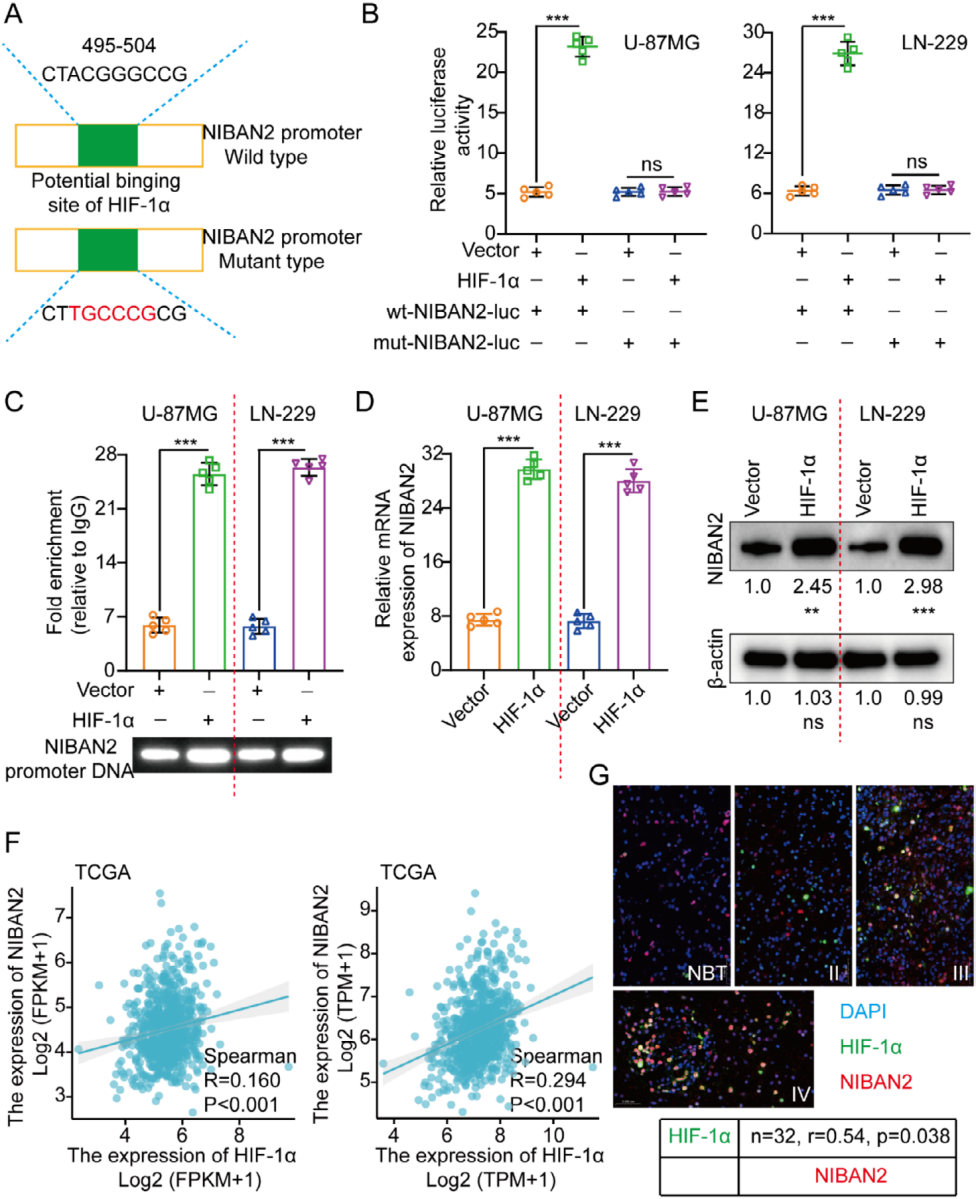
NIBAN2 was a direct target of HIF‐1α. (A) In accordance with the potential binding site of HIF‐1α in the *NIBAN2* promoter, wild‐type and mutant luciferase vectors were created. (B) To test luciferase activity, U‐87MG/LN‐229 cells were transfected with wild‐type or mutant luciferase vectors and co‐transfected with expression plasmids that included empty and HIF‐1α vectors. (C) To investigate the involvement of HIF‐1α in regulating genes, chromatin immunoprecipitation experiments were conducted, utilizing IgG as an internal control to ensure specificity. PCR was employed for the amplification of DNA associated with HIF‐1α using primers targeting the *NIBAN2* promoter. (D, E) NIBAN2 expression was assessed using qRT‐PCR and WB with artificially introduced HIF‐1α. (F) TCGA database outcomes indicated a positive relationship between HIF‐1α and NIBAN2 levels. (G) A positive correlation between HIF‐1α and NIBAN2 was observed through immunofluorescence staining and immune scoring in clinical samples. Ns, not significant.

Hypoxia is an important environmental condition of gliomas that promotes invasion, metastasis, and malignancy [20, 21, 22]. Transcription factors bind specific DNA sequences to regulate gene expression. According to the JASPAR database [23], HIF‐1α binds to the *NIBAN2* gene promoter. The regulation of *NIBAN2* transcription by HIF‐1α was investigated by introducing luciferase vectors comprising either wild‐type or mutant *NIBAN2* promoters into U‐87MG and LN‐229 cells (Figure 7A). As shown by the increased luciferase activity, HIF‐1α overexpression enhanced the activity of the WT *NIBAN2* promoter, but did not affect the activity of the mutant *NIBAN2* promoter (Figure 7B). Chromatin immunoprecipitation (ChIP) assays confirmed HIF‐1α binding to the *NIBAN2* promoter (Figure 7C), whereas HIF‐1α upregulation elevated NIBAN2 expression levels (Figure 7D,E). Analysis of TCGA data and IF staining further confirmed their positive correlation in glioma tissues (Figure 7F,G).

## 4 Discussion

Gliomas originate from glial cells that support and protect neurons in the CNS. Gliomas are divided into different types based on the origin of the cells (astrocytomas, ependymomas, oligodendrogliomas) and the degree of malignancy (low‐ or high‐grade) [23]. High‐grade gliomas, including GBM, are the most prevalent malignant brain tumors, with increased invasiveness and a poor prognosis [24]. The pathogenesis of glioma is complex and involves multiple factors, such as gene mutations, changes in the tumor microenvironment, and metabolic reprogramming. The management of gliomas predominantly involves surgical resection, radiation, and chemotherapy; however, their significant invasiveness and elevated posttreatment recurrence rate typically result in a brief survival duration [25, 26]. Therefore, an in‐depth exploration of new treatment strategies, including immunotherapy, targeted therapy, and gene therapy, is important for improving treatment efficacy and patient prognosis.

NIBAN2 is a cytoplasmic protein with functional domains that enable it to interact with other proteins and participate in intracellular signal transduction and regulation. Although relatively little research has been conducted on NIBAN2, some studies have revealed its potential roles in cellular functions and biological processes. Yu et al. reported that NIBAN2 regulates the cellular responses to oxidative stress and other forms of environmental stress. This may be associated with signaling pathways that regulate cell survival, such as the p53 pathway [3]. Evans et al. reported that NIBAN2 may be involved in the inhibition of cell death, thereby helping cells maintain their survival [6]. In some types of cancer, the expression of NIBAN2 may be associated with tumor invasiveness and metastasis. NIBAN2 may affect tumor behavior by controlling the aggressiveness of tumor cells [5]. NIBAN2 expression is upregulated or downregulated in many tumors, and these changes may affect the biological characteristics of the tumors. Pan‐Cancer Database analysis also showed that NIBAN2 was significantly overexpressed in most tumors (Figure S9). For example, some studies have found that NIBAN2 shows different expression patterns in gastric and liver cancers, which may be closely associated with tumor progression [4]. Further investigations revealed that NIBAN2 may be an important regulatory factor in the tumor microenvironment by affecting cell proliferation, migration, and immune escape mechanisms [27]. Additionally, the expression of NIBAN2 in the nervous system has garnered interest, particularly in neurodegenerative diseases. NIBAN2 may function in the survival, functional maintenance, and stress response of nerve cells [28].

The JAK2/STAT3/MYC signaling pathway is a complex cell‐signaling network involving multiple key molecules that play essential roles in cell proliferation and survival, immune regulation, and tumorigenesis [15]. The interaction between JAK2, STAT3, and MYC forms a feedback regulatory network that has a core function in numerous biological processes, particularly in the occurrence and development of tumors [29, 30, 31]. JAK2 is a non‐receptor tyrosine kinase that functions in conjunction with cytokine receptors. Cytokines (such as IL‐6 and EPO) activate JAK2 after binding to their receptors. Upon activation, JAK2 phosphorylates the intracellular portion of the receptor, providing binding sites for downstream signaling molecules, such as STAT3 [32]. STAT3 is a transcription factor that is normally phosphorylated upon activation by JAK2, after which it is translocated from the cytoplasm to the nucleus. STAT3 regulates target gene transcription in the cell nucleus by binding to DNA, and it participates in several processes, including cell growth and survival and the immune response [33]. The JAK2/STAT3 pathway plays a key role in the aggressiveness of several malignant tumors. Sustained activation of STAT3 can promote tumor cell growth and survival and help tumors escape immune surveillance [34]. MYC is a member of a family of transcription factors that includes c‐Myc, N‐Myc, and L‐Myc. Among these, c‐Myc is the most studied. MYC participates in cell proliferation, metabolism, differentiation, apoptosis, and other processes, and has an important function in many types of cancer [35]. It regulates the transcription of a large number of genes by interacting with other transcription factors or coactivators and is involved in processes such as the cell cycle, metabolism, apoptosis, and transformation. MYC is the main regulator of cell proliferation. It promotes rapid cell division by promoting cell cycle progression and the activation of metabolic pathways. MYC is a driving factor in many tumors, especially in the early stages. Overexpression of MYC is often closely related to the malignant transformation of cells and the formation of tumors [36, 37]. In tumor cells, continuous stimulation of the JAK2/STAT3 signaling pathway promotes MYC expression and enhances the effects of MYC on cell proliferation and metabolism, promoting the proliferation, survival, and invasion of tumor cells. Abnormal stimulation of the JAK2/STAT3/MYC pathway promotes tumor growth, metastasis, and drug resistance [30, 38]. Therapeutic strategies targeting JAK2, STAT3, and MYC are becoming popular research topics for anticancer treatments. Researchers are developing JAK2 inhibitors (such as ruxolitinib), STAT3 inhibitors, and MYC inhibitors as new treatment options for cancer patients [31, 38].

HIF‐1α is an essential transcription factor that has a core function in the adaptation of cells to hypoxic environments. It controls the expression of multiple genes that regulate cell metabolism, proliferation, apoptosis, angiogenesis, and other processes to cope with hypoxic environments [39]. HIF‐1α plays a role in physiological processes and has important clinical significance in numerous pathological states, including tumors and cardiovascular and neurodegenerative disorders. The stability and activity of HIF‐1α are strictly controlled by intracellular oxygen levels. Under a normoxic environment, HIF‐1α shows instability and is rapidly degraded through interactions with a variety of enzymes. However, in a hypoxic environment, HIF‐1α stability is enhanced, allowing it to exert its effects [40, 41]. In tumor cells, HIF‐1α is often in a state of continuous activation and can remain stable even in an oxygen‐sufficient environment. This abnormal HIF‐1α activation helps tumor cells grow in a hypoxic environment and enhances their resistance to treatment. HIF‐1α promotes the occurrence, development, and metastasis of tumors by promoting angiogenesis, accelerating metabolic changes, and supporting the survival of tumor cells [42]. The function of HIF‐1α in various disorders makes it a potential therapeutic target [43]. Modulating HIF‐1α activity or expression may become a new therapeutic strategy for various disorders, including tumors, cardiovascular diseases, and neurodegenerative diseases [44, 45].

**FIGURE 8:**
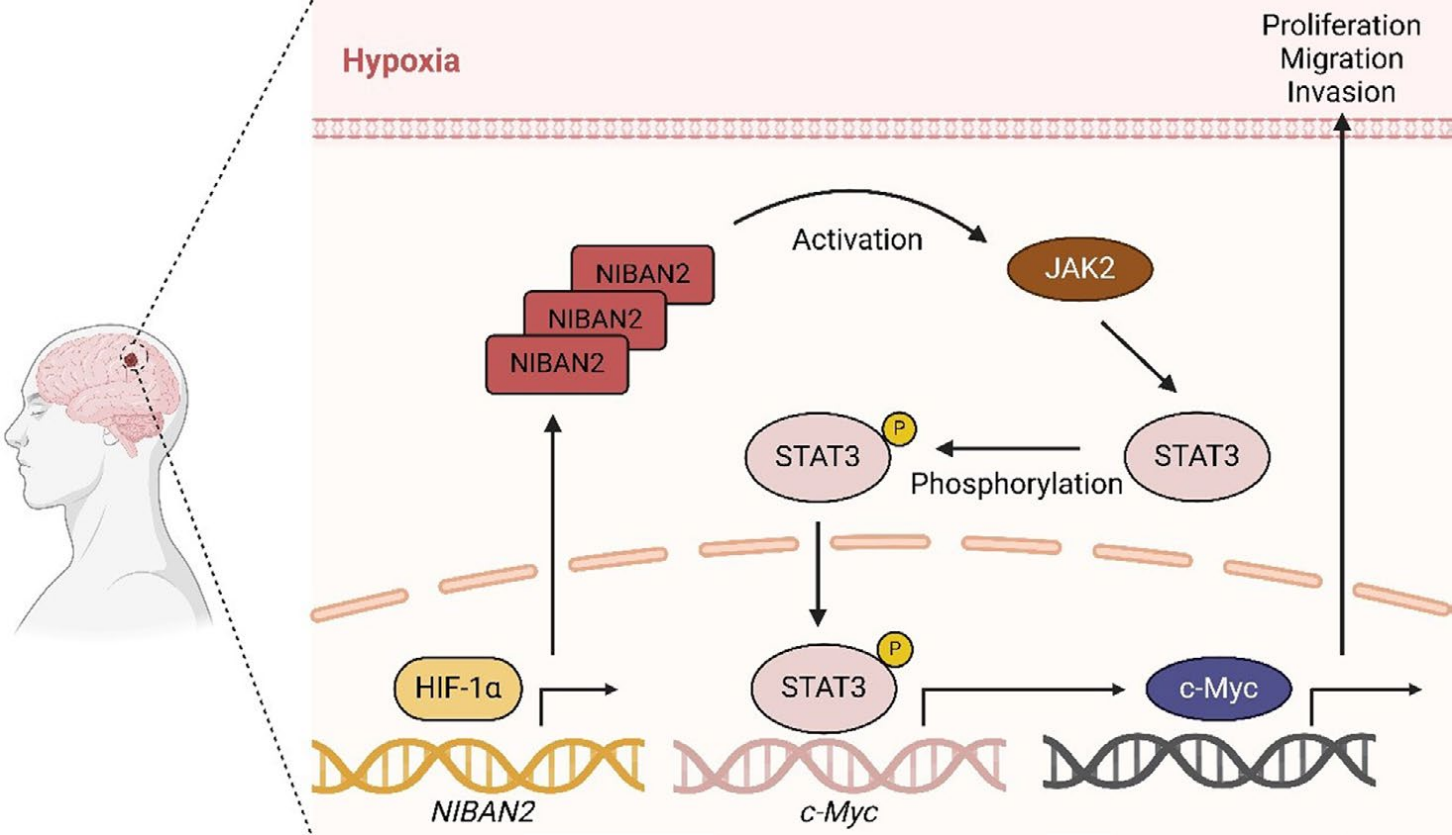
Schematic diagram of the mechanism. NIBAN2 is highly expressed in gliomas and its levels are inversely correlated with the patient's prognosis. Mechanistically, NIBAN2 activates the JAK2–STAT3 signaling pathway, thereby upregulating the protein levels of c‐Myc, and HIF‐1α in the local hypoxic microenvironment of the tumor directly targets NIBAN2 and upregulates the protein expression level of NIBAN2.

Our results showed that NIBAN2 was specifically overexpressed in gliomas and its expression level closely correlated with prognosis. Mechanistically, HIF‐1α targeted NIBAN2 to upregulate it, thereby promoting glioma progression by stimulating the JAK2/STAT3/c‐Myc signaling axis (Figure 8). This emphasizes the potential of NIBAN2 as a therapeutic target for glioma and suggests that further studies are necessary to elucidate its specific molecular mechanisms and evaluate its therapeutic relevance. Of course, our study also has certain limitations. For example, the precise molecular mechanisms by which NIBAN2 activates JAK2, as well as the specific process by which p‐STAT3 further regulates c‐Myc expression, remain to be elucidated and require further experimental validation.

## Author Contributions

**Zhi‐ming Chen:** conceptualization, writing – original draft, project administration. **Lei Mou:** conceptualization, writing – original draft. **Yi‐heng Pan:** data curation. **Chi Feng:** data curation. **Jun Liu:** data curation. **Jing‐jing Zhang:** data curation. **Chang‐Xiang Yan:** conceptualization, writing – original draft, visualization, data curation.

## Ethics Statement

The Ethical Board at Taihe Hospital approved this investigation.

## Consent

The authors have nothing to report.

## Conflicts of Interest

The authors declare no conflicts of interest.

## Supporting information


**Table S1:** Related antibodies involved.
**Table S2:** The specific shRNAs sequences utilized in this study.
**Table S3:** The Primer sequences in this study.
**Figure S1:** NIBAN2 bioinformatics analysis results.
**Figure S2:** Functional experiments of NIBAN2.
**Figure S3:** NIBAN2 prevents apoptosis and encourages the progression of the cell cycle.
**Figure S4:** NIBAN2 initiates the JAK2/STAT3 signaling pathway.
**Figure S5:** A positive correlation exists between NIBAN2 and molecules related to the JAK2/STAT3/MYC signaling pathway.
**Figure S6:** NIBAN2 enhances progression by triggering the JAK2/STAT3 signaling pathway.
**Figure S7:** NIBAN2 enhances glioma development by activating the JAK2‐STAT3‐c‐Myc signaling pathway.
**Figure S8:** NIBAN2 promotes glioma progression through c‐Myc.
**Figure S9:** Pan‐Cancer Database analysis results. Pan‐Cancer Database analysis also showed that NIBAN2 was significantly overexpressed in most tumors.

## Data Availability

The data that support the findings of this study are available from the corresponding author upon reasonable request.
