# Supplementary material for: NIBAN2 Stimulates Glioma Growth by Regulating the JAK2/STAT3/c‐Myc Pathway
Source: Cancer Med. 2025 Sep 13;14(18):e71239. doi: 10.1002/cam4.71239 (PMC12432436; doi:10.1002/cam4.71239)
Supplement: Supplementary file 1 — Table S1: Related antibodies involved. Table S2: The specific shRNAs sequences utilized in this study. Table S3: The Primer sequences in this study. Figure S1: NIBAN2 bioinformatics analysis results. Figure S2: Functional experiments of NIBAN2. Figure S3: NIBAN2 prevents apoptosis and encourages the progression of the cell cycle. Figure S4: NIBAN2 initiates the JAK2/STAT3 signaling pathway. Figure S5: A positive correlation exists between NIBAN2 and molecules related to the JAK2/STAT3/MYC signaling pathway. Figure S6: NIBAN2 enhances progression by triggering the JAK2/STAT3 signaling pathway. Figure S7: NIBAN2 enhances glioma development by activating the JAK2‐STAT3‐c‐Myc signaling pathway. Figure S8: NIBAN2 promotes glioma progression through c‐Myc. Figure S9: Pan‐Cancer Database analysis results. Pan‐Cancer Database analysis also showed that NIBAN2 was significantly overexpressed in most tumors. [file CAM4-14-e71239-s001.docx]

**NIBAN2 Stimulates Glioma Growth by Regulating the JAK2/STAT3/c-Myc Pathway**

Zhi-ming Chen^1,2*^, Lei Mou^3*^, Yi-heng Pan^3^, Chi Feng^3^, Jun Liu^3^, Jing-jing Zhang^4#^, Chang-Xiang Yan^1#^

**Materials and Methods**

**Cignal finder cancer 10-pathway reporter array**

All procedures were conducted in accordance with the manufacturer's instructions for the reagents. Cells were resuspended and subsequently plated into 96-well plates in conjunction with luciferase reporters designed to target prevalent cancer pathways. Following incubation, luciferase activity was measured.

**Real-time quantitative RT-PCR (qRT-PCR)**

Total RNA was isolated from cellular and tissue samples utilizing TRIzol and TRIzol LS reagents (Life Technologies). MicroRNAs (miRNAs) were subsequently reverse transcribed employing the Mir-X™ miRNA First-Strand Synthesis Kit (Clontech, Mountain View, CA, USA). Quantitative real-time polymerase chain reaction (qRT-PCR) was performed using the SYBR Green PCR Master Mix (Takara, Shiga, Japan) alongside primers detailed in Table S3. Messenger RNA (mRNA) expression levels were quantified using the 7500 Fast Real-Time PCR System (Applied Biosystems, Foster City, CA, USA), with glyceraldehyde 3-phosphate dehydrogenase (GAPDH) utilized as the internal reference gene.

**Table S1: Related antibodies involved**

| Name | Description |
| --- | --- |
| Anti-FAM129B antibody | ab224116 (Abcam, Cambridge, USA) |
| Anti-β-Actin antibody | #8457 (Cell Signaling Technology, Beverly, MA, USA) |
| Anti-JAK1 antibody | ab133666 (Abcam, Cambridge, USA) |
| Anti-p-JAK1 antibody | ab138005 (Abcam, Cambridge, USA) |
| Anti-JAK2 antibody | ab108596 (Abcam, Cambridge, USA) |
| Anti-p-JAK2 antibody | ab32101 (Abcam, Cambridge, USA) |
| Anti-STAT1 antibody | ab234400 (Abcam, Cambridge, USA) |
| Anti-p-STAT1 antibody | ab109461 (Abcam, Cambridge, USA) |
| Anti-STAT2 antibody | ab32367 (Abcam, Cambridge, USA) |
| Anti-p-STAT2 antibody | ab191601 (Abcam, Cambridge, USA) |
| Anti-STAT3 antibody | ab68153 (Abcam, Cambridge, USA) |
| Anti-p-STAT3 antibody | ab267373 (Abcam, Cambridge, USA) |
| Anti-c-Myc antibody | 10828-1-AP (Proteintech, Wuhan, China ) |
| Anti-HIF-1α antibody | 20960-1-AP (Proteintech, Wuhan, China ) |
| Anti-Ki-67 antibody | #9129 (Cell Signaling Technology, Beverly, MA, USA) |

**Table S2: The specific sh-RNAs sequences utilized in this study**

| **Gene** | **Primer** | **Sequence(5′-3′)** |
| --- | --- | --- |
| sh-NIBAN2#1 | forward | GTCCCTTCTTTGGATGTCCTT |
| sh-NIBAN2#2 | forward | GCAGAGCTGCTATGAGAAGAT |
| sh-c-Myc#1 | forward | TAATGATAACCAGAACTTGCT |
| sh-c-Myc#2 | forward | CACCACCAGCACACGGAACTA |

**Table S3: The Primer sequences in this study**

| **Gene** | **Primer** | **Sequence(5′-3′)** |
| --- | --- | --- |
| NIBAN2 | forward | CTGACGGAGTTCCTCCAGTTC |
|  | reverse | GAGGTTCCCCGAGAAGACGAT |
| c-Myc | forward | GGCTCCTGGCAAAAGGTCA |
|  | reverse | CTGCGTAGTTGTGCTGATGT |
| GAPDH | forward | GAGTCAACGGATTTGGTCGT |
|  | reverse | TTGATTTTGGAGGGATCTCG |

**Figure Legends**

**
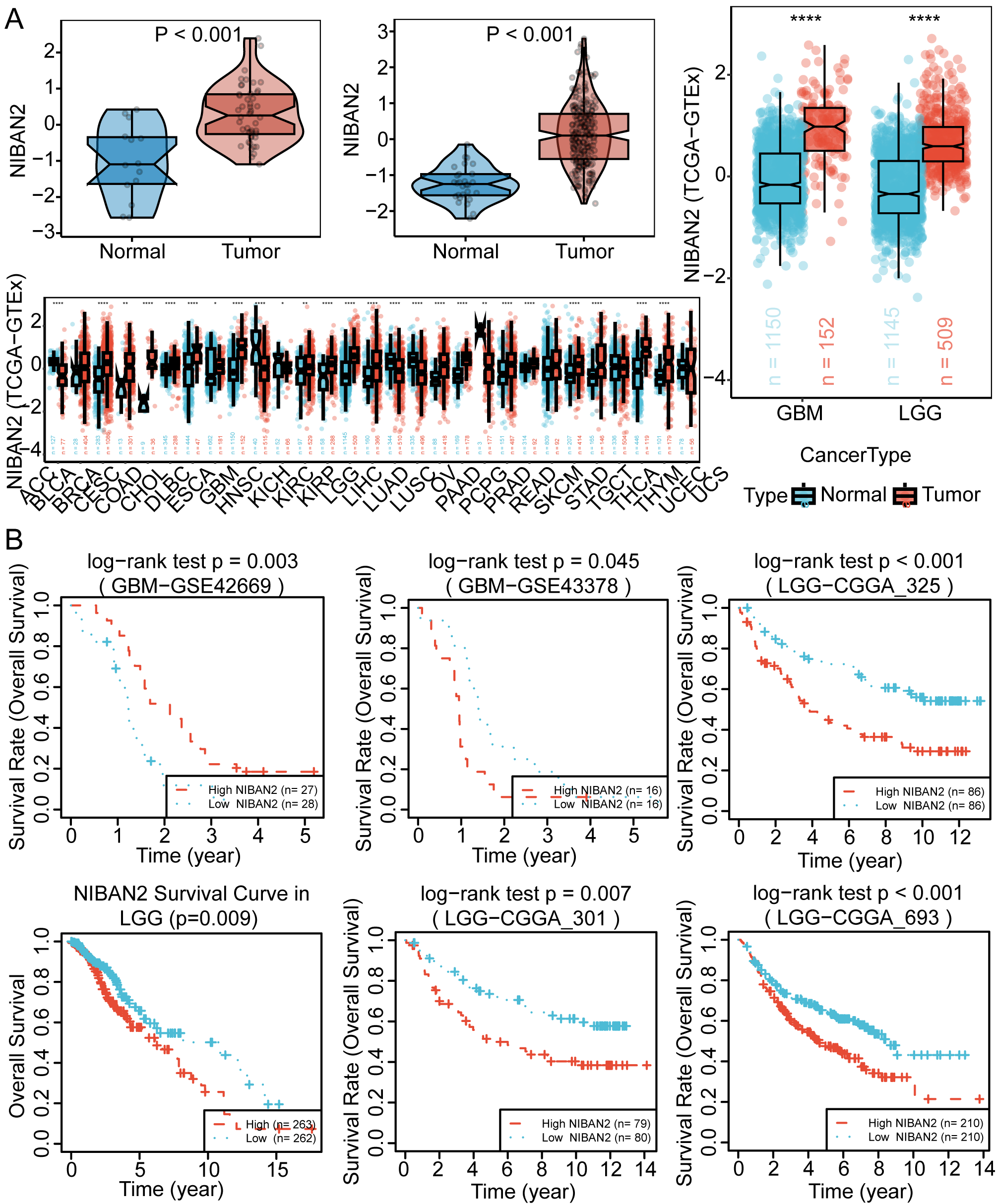
**

**Supplementary Figure 1: NIBAN2 Bioinformatics Analysis Results.**

**A.** Expression levels of NIBAN2 in different tissues. **B.** Association between NIBAN2 expression and patient prognosis.

**
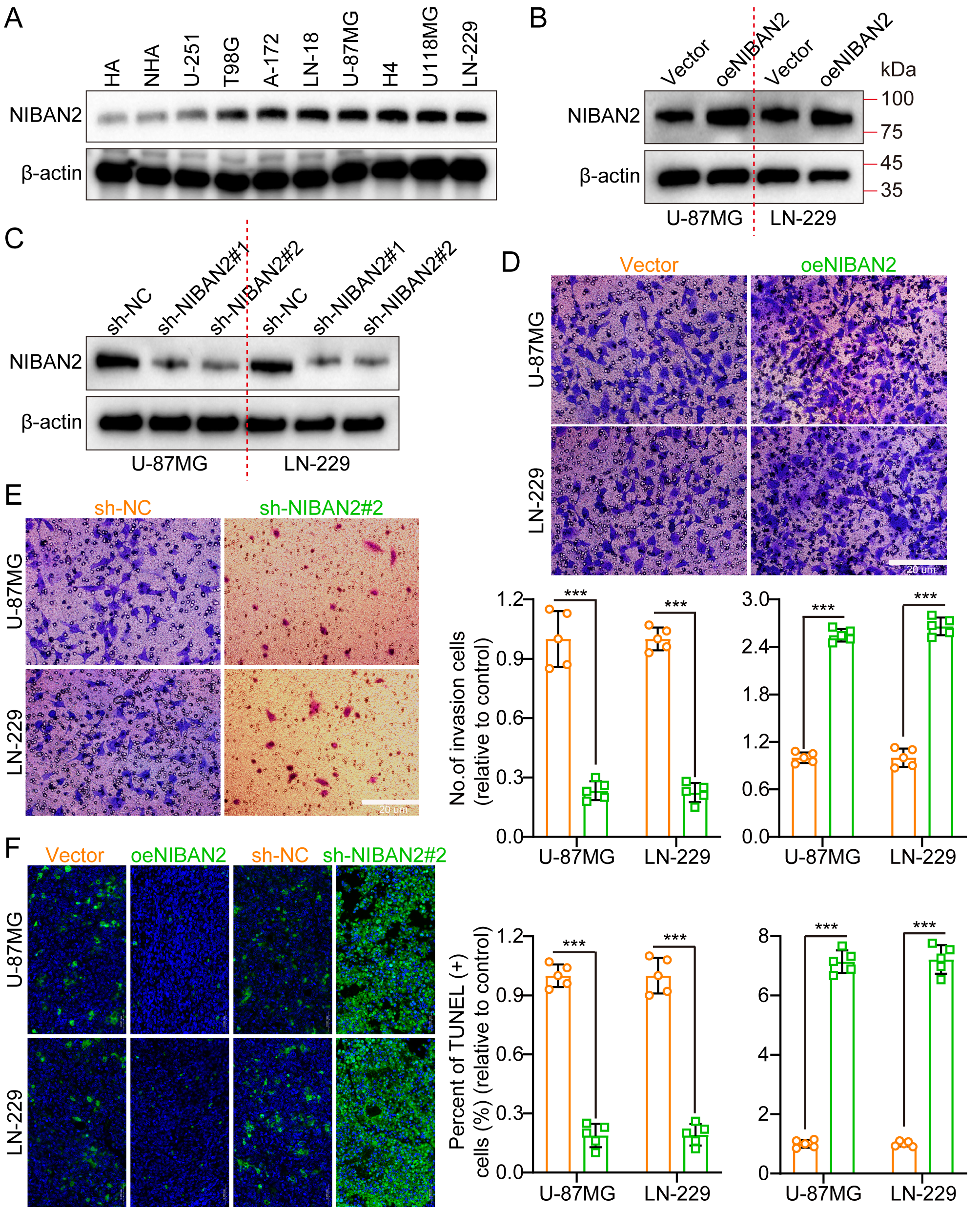
**

**Supplementary Figure 2: Functional experiments of NIBAN2.**

**A.** WB to assess NIBAN2 expression across HA, NHA, U-251, T98G, LN-229, A-172, LN-18, H4, U118MG, and U-87MG cell lines. **B-C.** Verification of NIBAN2 overexpression and knockdown effectiveness using Western Blot. **D.** Transwell invasion assays reveal that cell invasion is increased by NIBAN2 overexpression, n=5. Scale bars: 20 µm. **E.** Transwell invasion assays show that knocking down NIBAN2 reduced cell invasion, n=5. Scale bars: 20 µm. **F.** Representative images of TUNEL staining across different treatment groups, n=5. Scale bars: 0.050 mm. Statistical significance was tested using one-way ANOVA (Dunnett’s tests) for multiple comparisons and two-tailed t-tests. ****P* < 0.001.


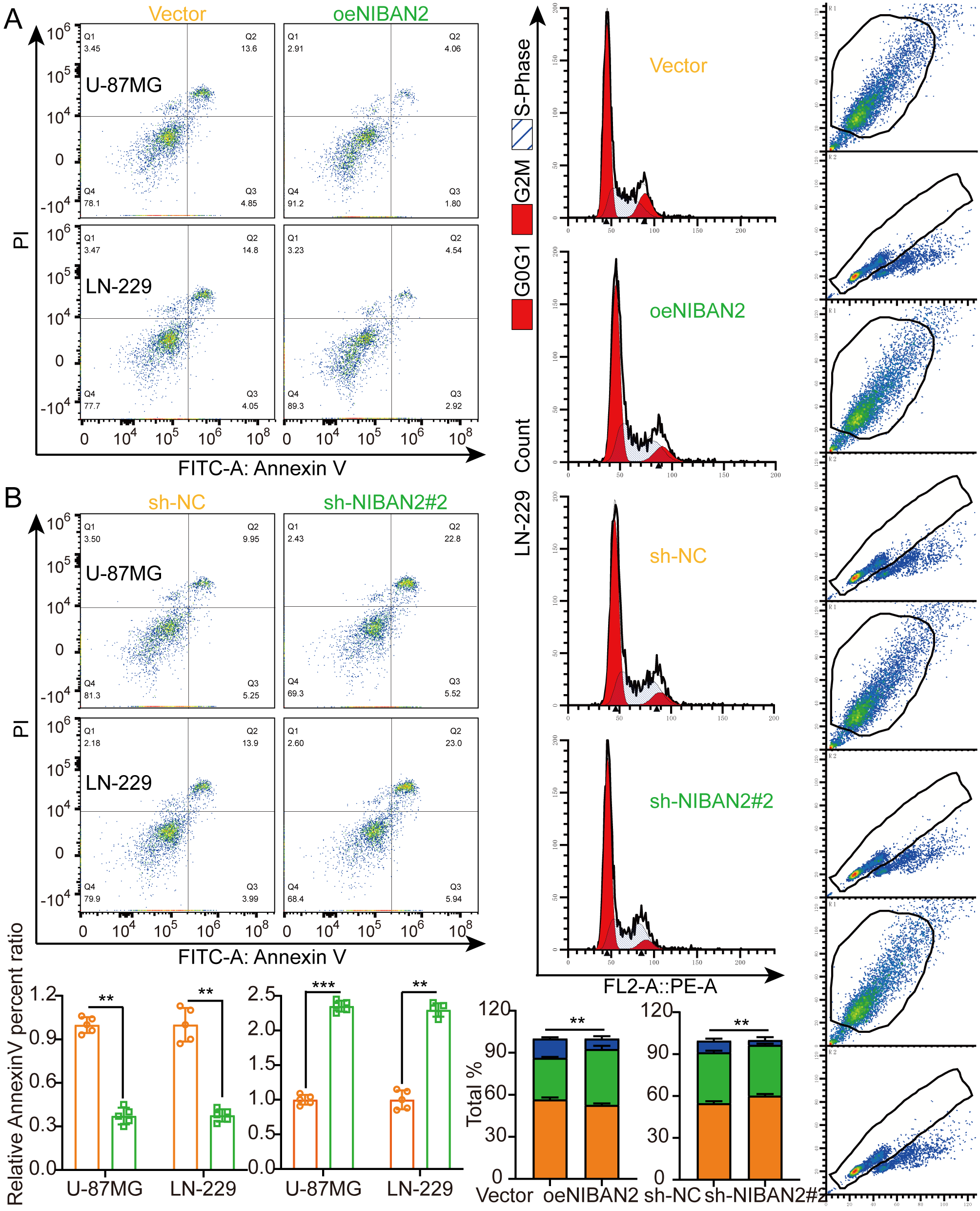


**Supplementary Figure 3: NIBAN2 prevents apoptosis and encourages the progression of the cell cycle.**

**A.** Flow cytometry results demonstrated that increasing NIBAN2 expression hindered apoptosis and encouraged cell cycle progression, n=5. **B.** Flow cytometry results indicated that silencing NIBAN2 enhanced apoptosis and slowed down cell cycle progression, n=5. Statistical significance was tested using one-way ANOVA (Dunnett’s tests) for multiple comparisons and two-tailed t-tests. ***P* < 0.01 and ****P* < 0.001.


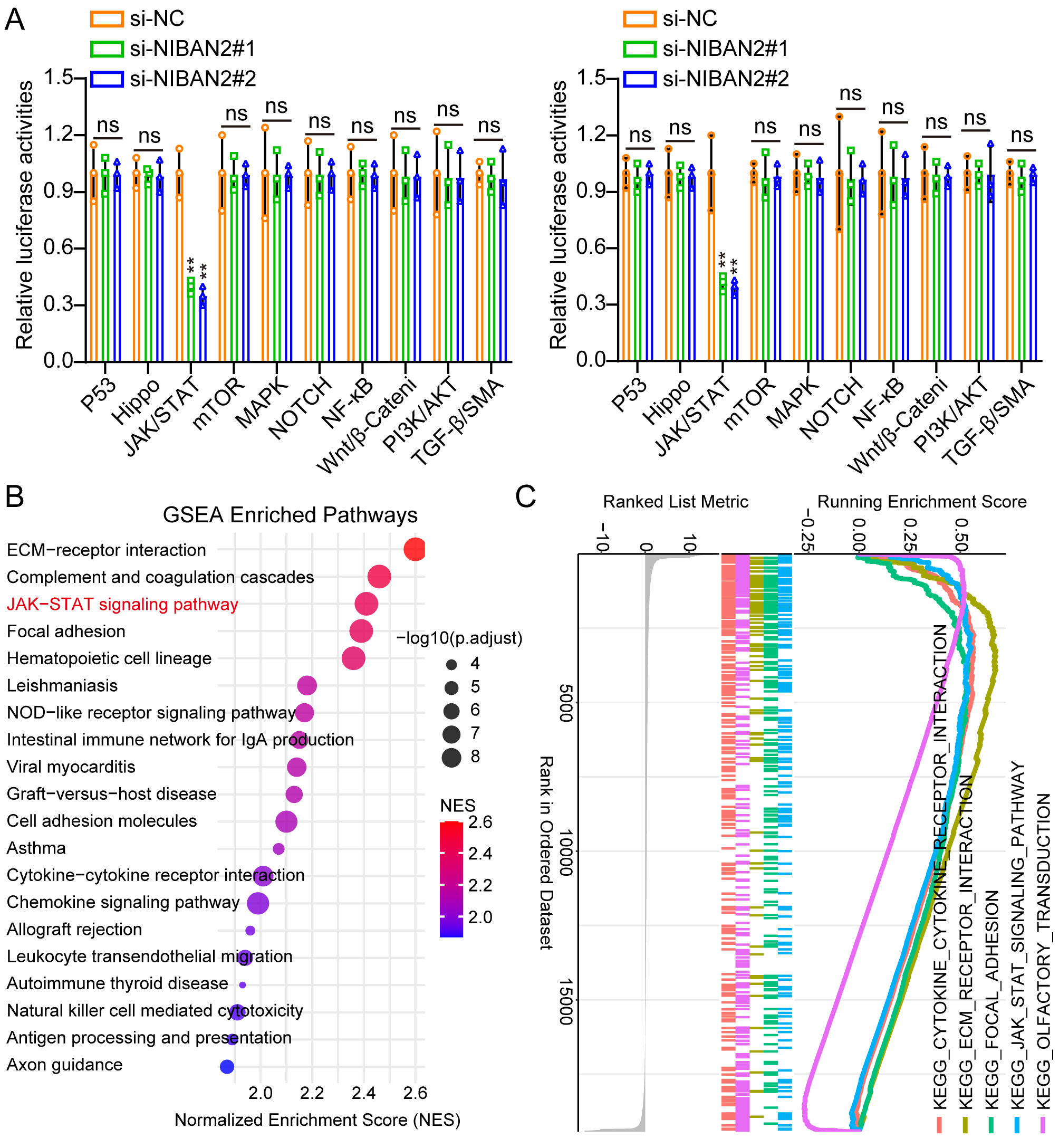


**Supplementary Figure 4: NIBAN2 initiates the JAK2/STAT3 signaling pathway.**

**A.** The Cignal Finder Cancer 10-Pathway Reporter Array revealed that knocking down NIBAN2 notably inhibited the JAK/STAT signaling pathway in U-87MG and LN-229 cells, which was not the case for other pathways. **B-C.** Gene Set Enrichment Analysis (GSEA) revealed a strong link between NIBAN2 and the JAK/STAT signaling pathway. Data are presented as Mean ± s.d from five independent experiments. Statistical significance was tested using one-way ANOVA (Dunnett’s tests) for multiple comparisons and two-tailed t-tests. ***P* < 0.01 and ****P* < 0.001. NS: No statistical difference.


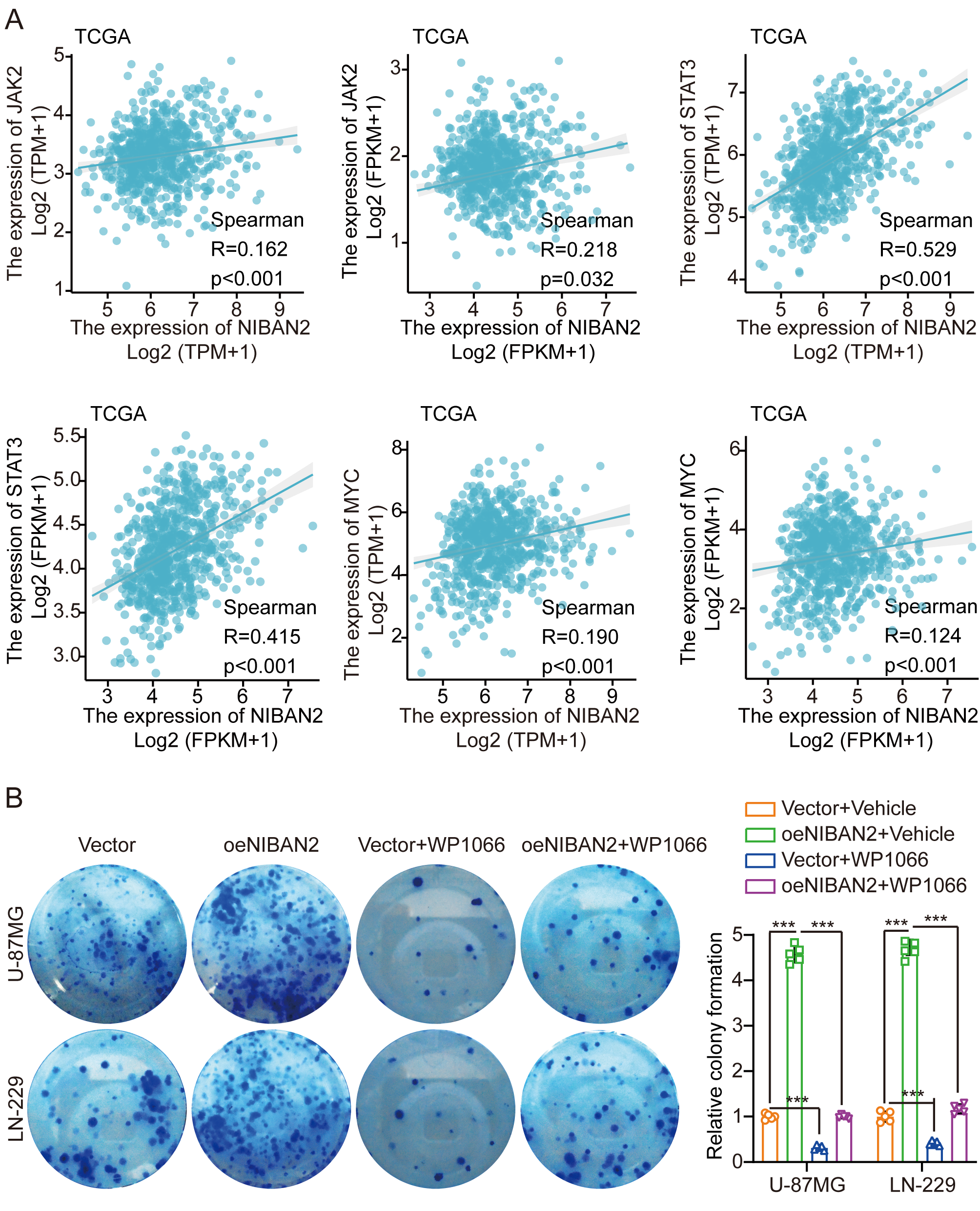


**Supplementary Figure 5: A positive correlation exists between NIBAN2 and molecules related to the JAK2/STAT3/MYC signaling pathway.**

**A.** The TCGA database analysis revealed a positive correlation between NIBAN2 and molecules associated with the JAK2/STAT3/MYC signaling pathway. **B.** A colony formation assay and histogram quantification were used to evaluate cell growth across different treatment groups. Data are presented as Mean ± s.d from five independent experiments. Statistical significance was tested using one-way ANOVA (Dunnett’s tests) for multiple comparisons and two-tailed t-tests. ****P* < 0.001.


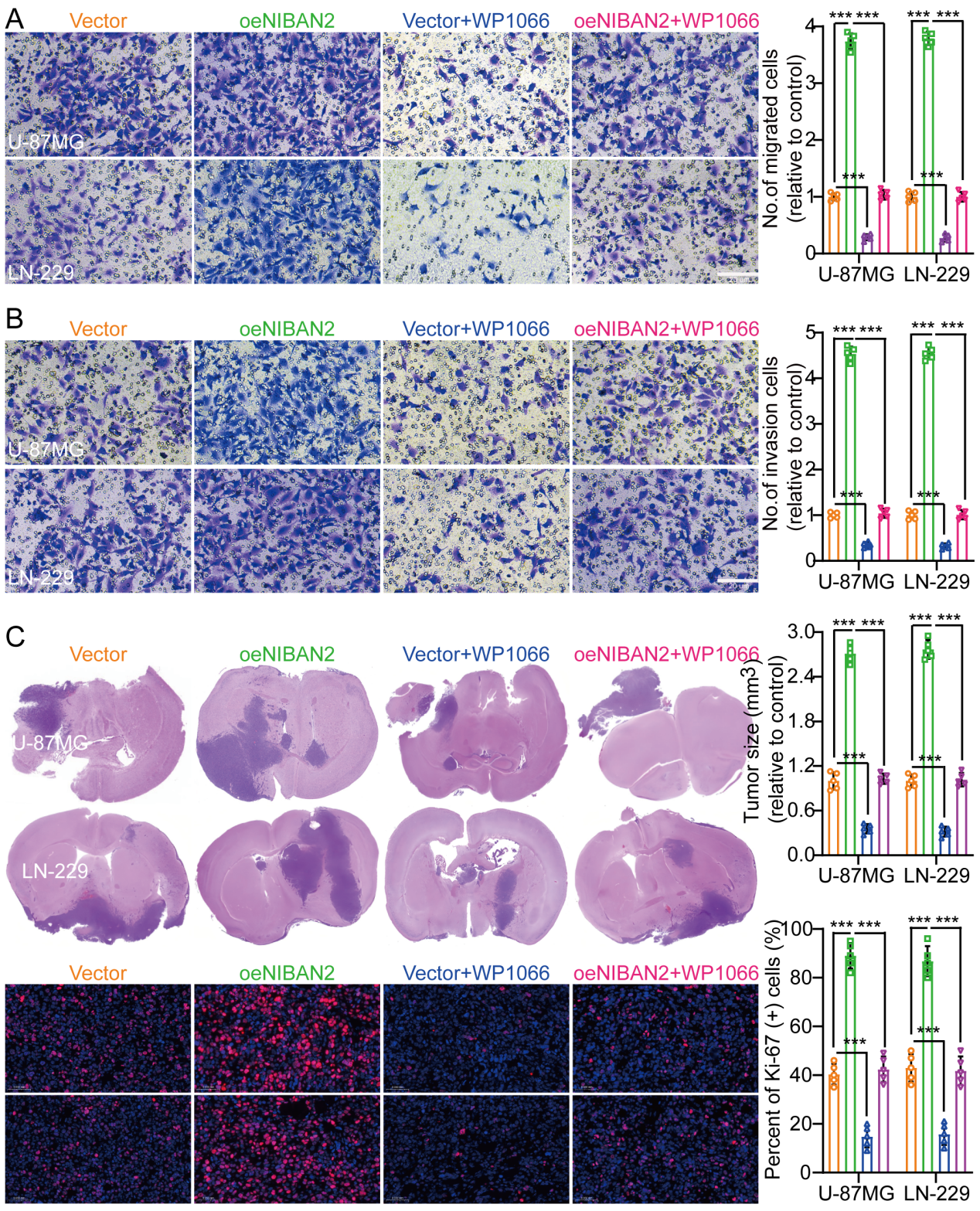


**Supplementary Figure 6: NIBAN2 enhances progression by triggering the JAK2/STAT3 signaling pathway.**

**A-B.** Malignant phenotype of cells across various treatment groups was assessed using a Transwell migration/invasion assay and histogram quantification. **C.** Representative images of Ki-67 staining and frozen sections of mouse brain tissue for each treatment group. Data are presented as Mean ± s.d from five independent experiments. Statistical significance was tested using one-way ANOVA (Dunnett’s tests) for multiple comparisons and two-tailed t-tests. ****P* < 0.001.


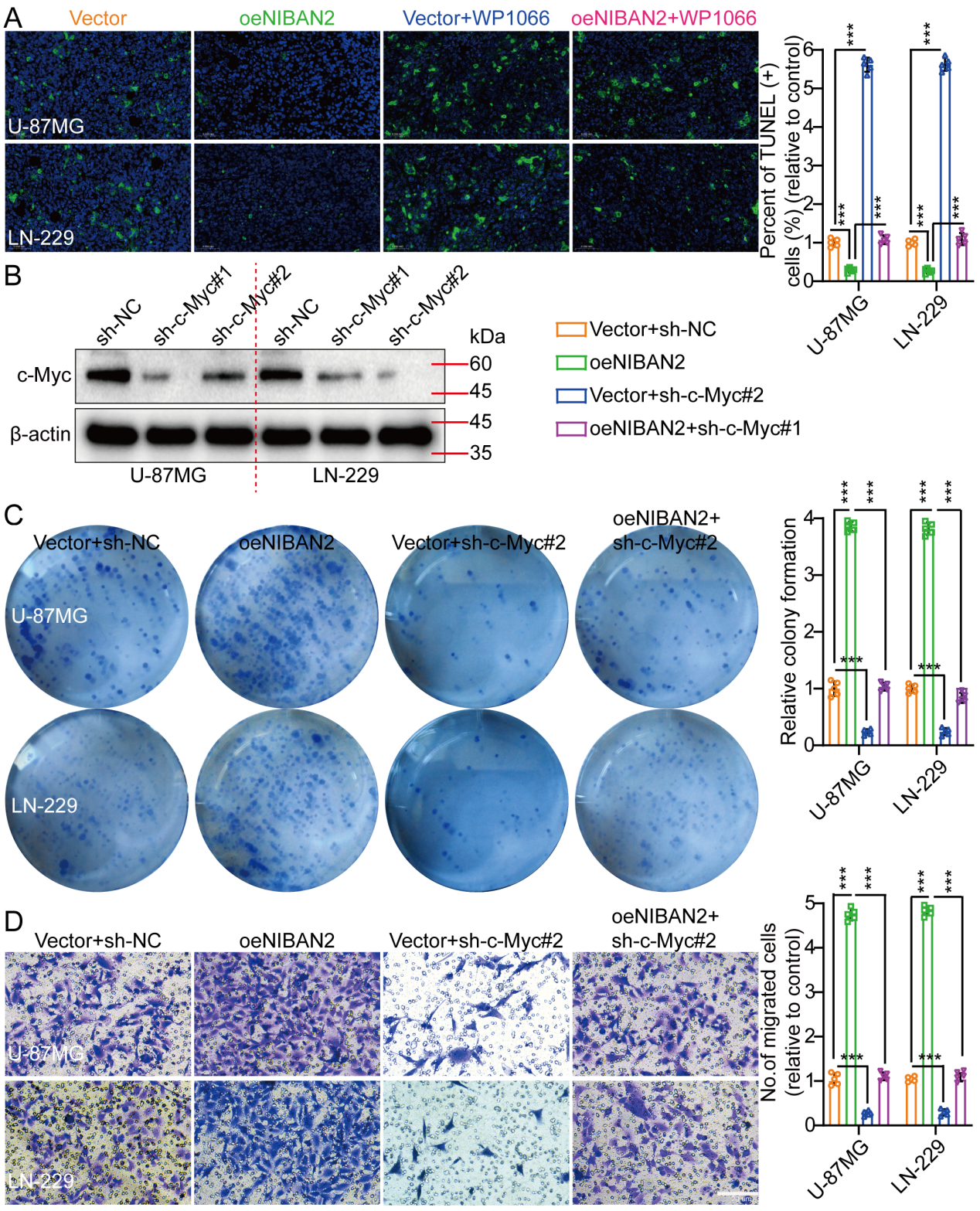


**Supplementary Figure 7: NIBAN2 enhances glioma development by activating the JAK2-STAT3-c-Myc signaling pathway.**

**A.** Representative pictures of TUNEL-stained at different treatment groups, n=5. Scale bars: 0.050 mm. **B.** Validation of c-Myc overexpression and knockdown efficiency via WB. **C.** Cell growth was assessed using a colony formation assay and histogram quantification across various treatment groups. **D.** Transwell migration assay and histogram quantification were used to evaluate cell growth across different treatment groups. Statistical significance was tested using one-way ANOVA (Dunnett’s tests) for multiple comparisons and two-tailed t-tests. ****P* < 0.001.


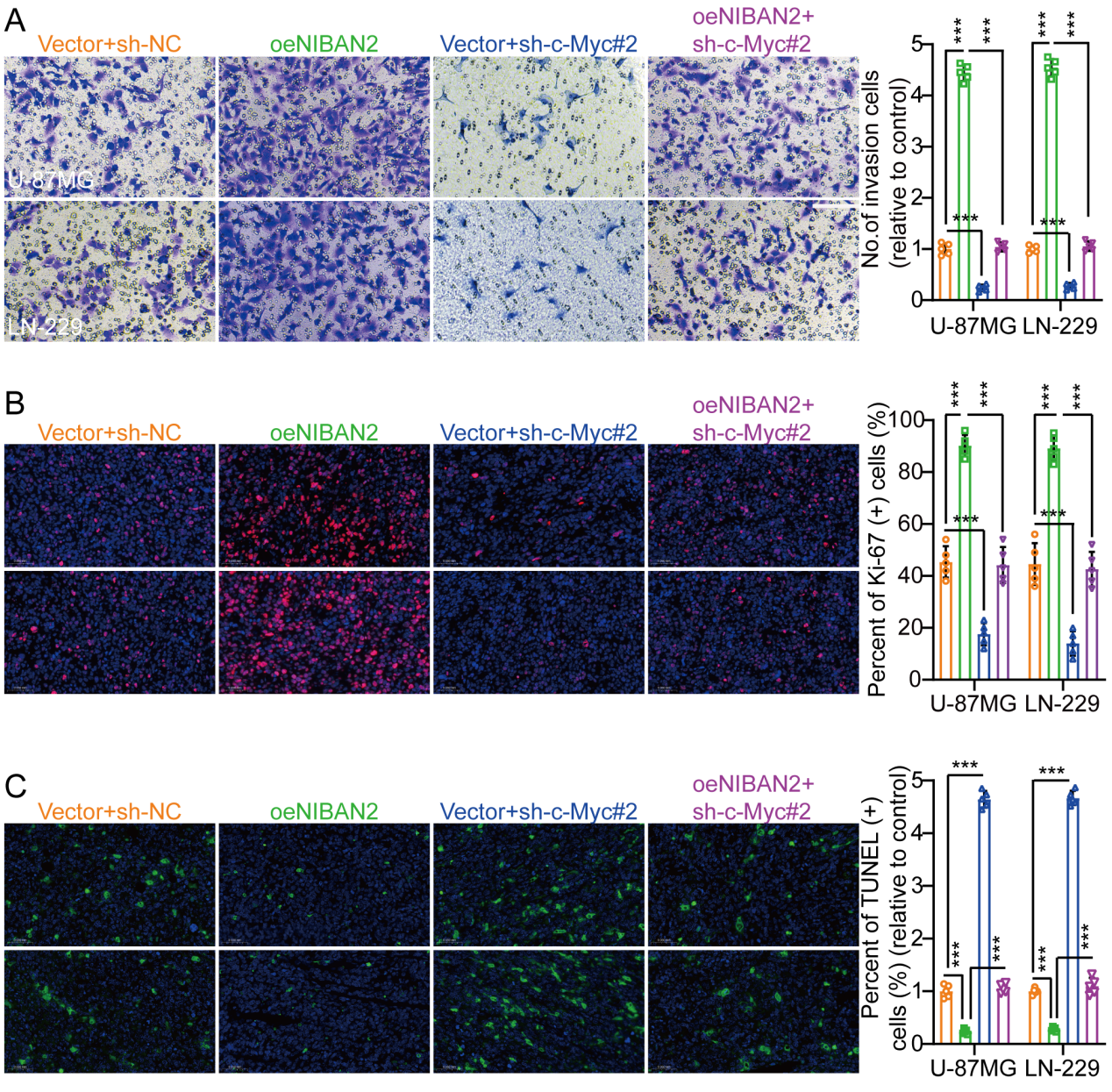


**Supplementary Figure 8: NIBAN2 promotes glioma progression through c-Myc.**

**A.** Transwell invasion assay and histogram quantification were used to evaluate cell growth across different treatment groups. **B.** Representative pictures of TUNEL-stained at different treatment groups, n=5. Scale bars: 0.050 mm. **C.** Representative pictures of TUNEL-stained at different treatment groups, n=5. Scale bars: 0.050 mm. Statistical significance was tested using one-way ANOVA (Dunnett’s tests) for multiple comparisons and two-tailed t-tests. ****P* < 0.001.


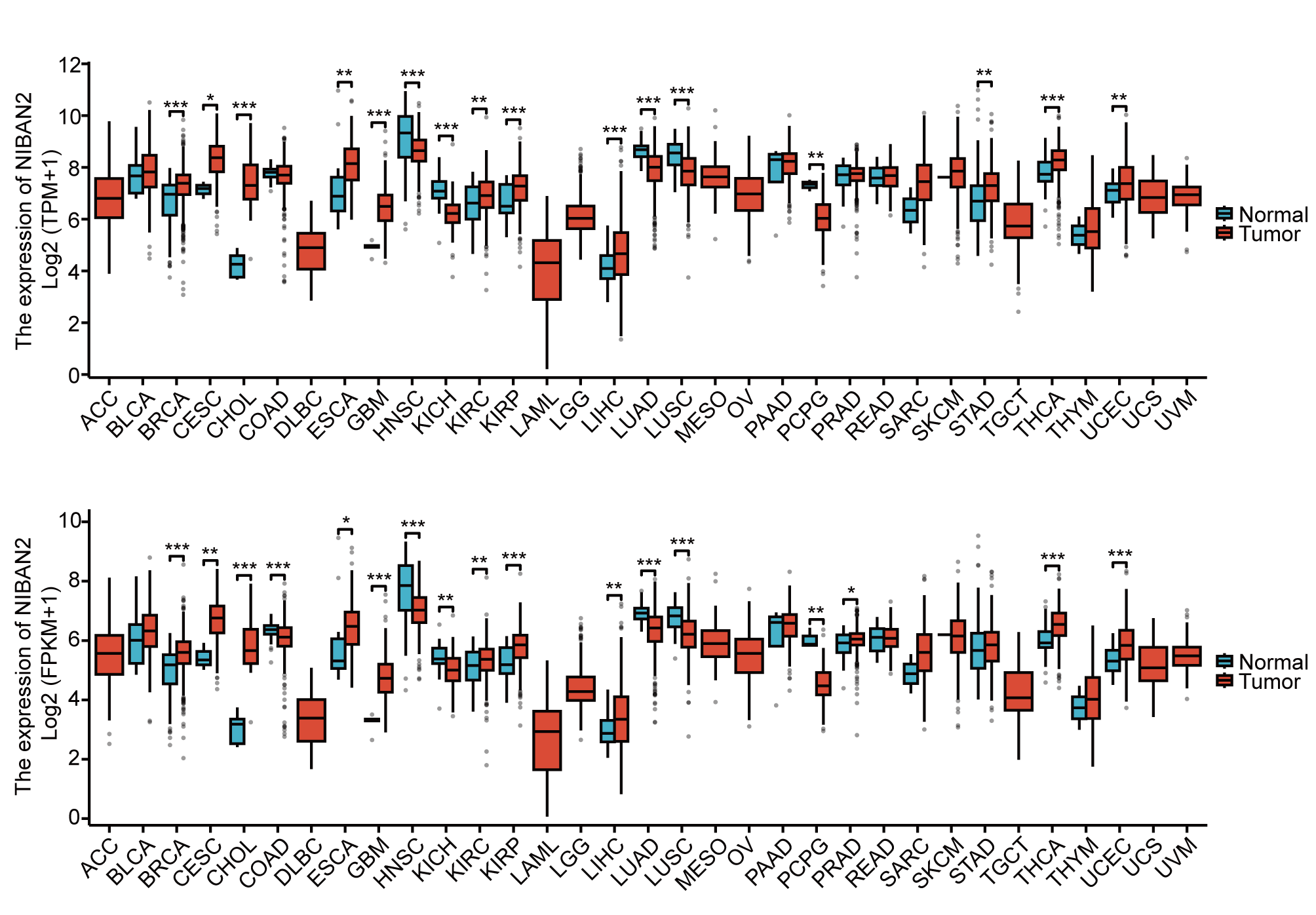


**Supplementary Figure 9: Pan-Cancer Database Analysis Results.** Pan-Cancer Database analysis also showed that NIBAN2 was significantly overexpressed in most tumors
